# Nanostructure and Collagen‐Stimulating Activity of Cationic Pentapeptide Lipopeptides

**DOI:** 10.1002/psc.70111

**Published:** 2026-06-19

**Authors:** Lucas R. de Mello, Valeria Castelletto, Jani Seitsonen, Ian W. Hamley

**Affiliations:** ^1^ School of Chemistry, Food Biosciences and Pharmacy University of Reading Reading UK; ^2^ Nanomicroscopy Center Aalto University Espoo Finland

## Abstract

The self‐assembly in aqueous solutions, cytocompatibility, and collagen production of lipopeptide C_16_–KTTKS and a variant with arginine substitution for lysine, C_16_–RTTRS, are investigated. C_16_–KTTKS is known commercially as Matrixyl and is used in cosmetic formulations as it can stimulate collagen production. The self‐assembly and conformations and collagen‐stimulating effects of the two lipopeptides in two salt forms, trifluoroacetate (TFA) and acetate, are compared. Lipopeptide C_16_‐KTTKS self‐assembles into nanotapes based on a multi‐bilayer stacking across a pH range pH 4–7 and micelles at pH 2, with little influence of the counterion. In contrast, C_16_‐RTTRS forms a substantial population of spherical micelles for the acetate salt for pH 2–7, but mainly nanotapes for the TFA salt for pH 4–7. Conditions for hydrogel formation by C_16_‐KTTKS were identified. Both lipopeptides show good cytocompatibility to fibroblasts at sufficiently low concentration. The two lipopeptides also stimulate collagen production in Human Dermal Fibroblasts (HDFa) at low concentration (0.0062 wt%). No significant effect of the counterion was noted on cell viability or collagen production. Our results suggest that the peptide sequence influences the pH‐dependent self‐assembly properties and that this can be modulated for certain lipopeptides by the nature of the counterions.

## Graphical abstract

The self‐assembly in aqueous solutions, cytocompatibility and collagen production of lipopeptide C_16_–KTTKS (C_16_: hexadecyl) and a variant with arginine substitution for lysine, C_16_–RTTRS, are compared.

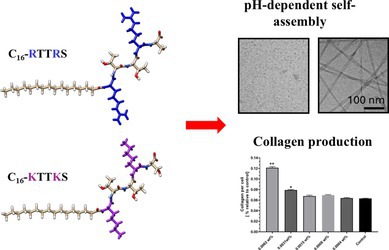

## 1 Introduction

Lipopeptides are bioconjugate molecules consisting of a peptide sequence, which can be endowed with a range of bioactivities, and a linked lipid tail. Bioactive peptides are usually short designed or natural sequences and show promise for applications in biomedicine and biotechnology [1, 2, 3, 4, 5, 6]. Lipidation is a natural posttranslational modification, although designed lipopeptides are typically created synthetically via simple coupling methods often via N‐terminal conjugation. The amphiphilicity that results from linking of hydrophilic peptides and hydrophobic lipid chains can induce self‐assembly, which in turn can have a profound impact on bioactivity [1, 6, 7, 8, 9, 10, 11, 12]. Self‐assembled structures include nanosheets, nanofibrils, nanotubes, micelles, and nanotapes, each with unique properties that can be beneficial for the intended effect of the lipopeptide. Lipopeptides designed for antimicrobial and other properties can show enhanced activity from micellar structures [10, 13], while lipopeptide fibrils can show better activity than bilayer nanotapes as shown for example in a recent study on lipopeptides bearing lysine‐rich tripeptides [12]. Lipopeptides can also form hydrogels with applications in tissue engineering and biotechnology [14, 15].

The self‐assembly of peptides and lipopeptides depends on many parameters, solution conditions including pH and concentration, presence of buffers, temperature, etc. Residual counterions from solid‐phase peptide synthesis (SPPS), such as trifluoracetate (TFA) or acetate may impact both self‐assembly and cytocompatibility of a peptide sequence. TFA may interfere with the hydrogen bonding network, and also induce cytotoxicity or even reduce cell proliferation, while acetate salts tend to be better tolerated than TFA and have been approved by the FDA as certified pharmaceutical salts for drugs [16, 17, 18].

The conjugation of lipid chains to bioactive peptides is important in cosmetics or wound healing, since in addition to improved stability, the lipidation may improve compatibility with cell membranes and enhance penetration into tissue. One lipopeptide widely used in the cosmetic market couples the KTTKS pentapeptide and a hexadecyl lipid giving C_16_‐KTTKS within the Matrixyl family, developed by Sederma. The KTTKS pentapeptide is derived from a pro‐peptide from Human type I collagen [19, 20]. Both peptide KTTKS [19] and the lipopeptide C_16_‐KTTKS [21] are able to promote type I collagen production in vitro. It is also suggested that C_16_‐KTTKS can reduce the appearance of facial wrinkles and may help in closing skin wounds faster [22, 23, 24]. Topical application of lipopeptides requires transport of the active across the stratum corneum, and Matrixyl lipopeptides were developed to improve delivery across the epidermis [22, 23]. Our group showed that at native pH, C_16_‐KTTKS self‐assembles into highly extended nanotapes, based on a bilayer packing of β‐sheets of the lipopeptide molecules [25]. The self‐assembly of this lipopeptide is pH dependent (at 20°C) and we found that the TFA salt of C_16_‐KTTKS forms micelles at low pH [26]. It is also temperature dependent, forming spherical micelles at high temperature (at native pH, for the TFA salt) in contrast to the nanotapes observed at 20°C [27].

We also examined the self‐assembly of the related lipopeptides C_14_‐KTTKS and C_18_‐KTTKS with distinct lipid chain lengths [28], as well as that of a lipopeptide with shorter peptide sequence C_16_‐KT (along with other lipopeptides relevant to skincare applications) [29]. A homologue bearing anionic glutamic acid residues instead of cationic lysine residues, C_16_‐ETTES [30] was introduced by our group and its self‐assembly into nanotapes as well as its co‐assembly with C_16_‐KTTKS was studied [30]. Lipopeptide C_16_‐ETTES was also used as a diluent in mixtures with a lipopeptide bearing an RGD integrin‐binding motif, and the structure and conformation of the co‐assemblies was studied in detail [31]. The mixtures were shown to have promising cell adhesion and proliferation properties cell culture and tissue engineering [32]. They could also form stable coatings on oriented hydrophobic substrates, creating aligned extracellular matrix [32]. The self‐assembly and bioactivity of a conjugate of KTTKS to lipoic acid has recently been examined by our group. The lipoyl group at the N terminus enables redox activity (chemical and photoinduced) through reduction of the dilsulfide bond [33]. We found that lipoyl‐KTTKS has excellent cytocompatibility, stimulates collagen production and enhances the rate of cell coverage in a simple in vitro scratch assay of “wound healing” [33]. We have also designed lipopeptides bearing one or two cycloalkane chains (cycloheptadecyl or cyclododecyl) conjugated to KTTKS and self‐assembly and bioactivity were examined, including in vivo wound healing studies [34]. The conjugate containing two cyclododecyl lipids showed great promise in terms of collagen deposition and wound healing properties including promotion of vascularization. Analogues with C‐terminal residue substitutions (C_16_‐KTTKY) and glutamic acid (C_16_‐KTTKE) have recently been reported, and the former shows a collagen‐stimulating effect in contrast to the latter [35]. In addition, the lipopeptides were found to stimulate *Staphylococcus epidermidis* growth, a microorganism very important for skin microbiota health. The collagen production activity of a derivative of KTTKS with N‐terminal ascorbyl unit has been compared with that of KTTKS and C_16_‐KTTKS [36]. A large series of KTTKS peptide derivatives with K/R substitutions, N‐terminal lipidation, and different terminal capping have recently been screened for cytotoxicity and proteolytic activity, the latter being relevant to degradation of the extracellular matrix [37]. Collagen biosynthesis was also assayed for selected peptides.

Here, we compare the self‐assembly, conformation, and cytocompatibility and collagen production bioactivity of C_16_‐KTTKS and C_16_‐RTTRS. The rationale behind exchanging lysine for arginine is that although both are cationic charged polar amino acids, the H‐bonding capacity of arginine (R) is higher than for lysine (K), due to the delocalized guanidinium side chain in R, which can influence self‐assembly [38, 39], and thus potentially bioactivity. There are also significant differences in the pKa values for the side chains of lysine (pKa 10.5) and arginine (pKa 12.5). The lysine side chain is also longer and less sterically hindered than that of arginine. Here, we compare the conformation and self‐assembly of both lipopeptides as a function of pH. We also explore the effect of counterions on self‐assembly, comparing the association behavior of acetate and TFA salts. Peptides and lipopeptides prepared by solid phase synthesis are most commonly prepared as TFA salts. The counterion can influence profoundly self‐assembly as shown, for example, for Lanreotide [40]. Trifluoroacetic acid is much more acidic (pKa = 0.2) than acetic acid (pKa 4.8), and this could influence the localization of the counterions around or within a self‐assembled structure and in turn could affect interactions especially electrostatic interactions, and hence impact the peptide/lipopeptide self‐assembly. Here, we show that this in fact does occur for C_16_‐KTTKS and C_16_‐RTTRS comparing acetate and TFA salts since different nanostructures are observed under certain pH conditions. Here, we also report that at higher concentrations C_16_‐KTTKS forms a stable hydrogel at pH 7, for which the mechanical properties were probed. TFA is also known to have effects in the bioactivity and cytocompatibility of a protein or peptide, triggering immune responses, even for example inhibiting the proliferation of osteoblasts [41, 42]. However, the data presented herein show little effect of the counterion on cytocompatibility or collagen production of dermal fibroblasts for C_16_‐KTTKS and C_16_‐RTTRS.

Bioactivity was examined through MTT [3‐(4,5‐dimethylthiazol‐2‐yl)‐2,5‐diphenyltetrazolium bromide] assays to determine cytotoxicity and collagen production was assayed using the picrosirius red staining method (sensitive to type I and type III collagen).

## 2 Methods

### 2.1 Reagents and Sample Preparation

Lipopeptides were purchased from Peptide Synthetics (Peptide Protein Research, Fareham, United Kingdom) and supplied as acetate or TFA salts. The molar masses measured by ESI‐MS are as follows: C_16_‐KTTKS 802.06 g mol^−1^ (802.87 g mol^−1^ expected) and C_16_‐RTTRS 858.08 g mol^−1^ (858.10 g mol^−1^ expected). The purity by HPLC (0.1% TFA in acetonitrile/water gradient) is 97.5% for both salts of C_16_‐KTTKS and 95.2% for both salts of C_16_‐RTTRS.

Solutions were prepared by dissolution in ultrapure water, with measured (Mettler Toledo FiveEasy pH meter with a Sigma‐Aldrich micro pH combination glass body electrode) native pH values for 1 wt% solutions as follows: C_16_‐KTTKS (TFA) pH 2.3, C_16_‐KTTKS (acetate) pH 4, C_16_‐RTTRS (TFA) pH 2.4, and C_16_‐RTTRS (acetate) pH 5.3. The pH of samples was adjusted using 100 mM NaOH or HCl. Samples intended for tissue and cell culture assays were incubated in DMEM/F12 and PBS for biological assays, both at pH = 7.4.

To prepare hydrogel samples for rheology, solutions containing 0.5 or 2 wt% of C_16−_KTTKS in citrate buffer at pH 7 were heated to 70°C in an ultrasonic bath until fully homogeneous and transparent to the naked eye. Then, the gels were vortexed while cooling down to room temperature. The 0.5‐wt.% sample was left to rest overnight at room temperature, to produce a soft gel, whereas the 2‐wt.% sample only took a few minutes to produce a self‐standing gel at room temperature.

### 2.2 Cryogenic‐TEM (Cryo‐TEM)

Imaging was carried out using a field emission cryo‐electron microscope (JEOL JEM‐3200FSC), operating at 200 kV. Images were taken in bright field mode and using zero loss energy filtering (omega type) with a slit width of 20 eV. Micrographs were recorded using a Gatan Ultrascan 4000 CCD camera. The specimen temperature was maintained at −187°C during the imaging. Vitrified specimens were prepared using an automated FEI Vitrobot device using Quantifoil 3.5/1 holey carbon copper grids with a hole size of 3.5 μm. Just prior to use, grids were plasma cleaned using a Gatan Solarus 9500 plasma cleaner and then transferred into the environmental chamber of a FEI Vitrobot at room temperature and 100% humidity. Thereafter, 3 μL of sample solution was applied on the grid, and it was blotted twice for 5 s and then vitrified in a 1/1 mixture of liquid ethane and propane at a temperature of −180°C. The grids with vitrified sample solution were maintained at liquid nitrogen temperature and then cryo‐transferred to the microscope.

### 2.3 Small‐Angle X‐Ray Scattering (SAXS)

SAXS experiments were performed on beamline B21 at Diamond (Didcot, United Kingdom) [43]. The sample solutions were loaded into the 96‐well plate of an EMBL BioSAXS robot and then injected via an automated sample exchanger into a quartz capillary (1.8 mm internal diameter) in the X‐ray beam. The quartz capillary was enclosed in a vacuum chamber, to avoid parasitic scattering. After the sample was injected into the capillary and reached the X‐ray beam, the flow was stopped during the SAXS data acquisition. More viscous samples (gel‐like in some cases) were measured in a custom‐made cell to hold gels between polyimide (Kapton) windows [44] or in capillaries, which were loaded into a holder in the beam path by hand. Beamline B21 operates with a fixed camera length (3.9 m) and fixed energy (12.4 keV). The images were captured using a PILATUS 2M detector. Data processing was performed using dedicated beamline software ScÅtter.

### 2.4 Circular Dichroism (CD) Spectroscopy

Far‐UV CD spectra were collected using a Chirascan spectropolarimeter (Applied Photophysics, Leatherhead, United Kingdom). Spectra were recorded from 180 to 400 nm. Samples were mounted in quartz cells with detachable windows, with 0.01 or 0.1 mm path length, depending on the concentration. CD spectra from the samples were corrected by water background subtraction. The CD spectra were smoothed using the built‐in Chirascan Software for data analysis. The residue of the calculation was chosen to oscillate around the average to avoid artifacts in the smoothed curve. CD data, measured in mdeg, were normalized to molar ellipticity using the molar concentration of the sample and the cell path length.

### 2.5 Fluorescence Spectroscopy

To investigate the critical aggregation concentration (CAC), fluorescence spectroscopy assays were performed using the fluorescent probe Thioflavin T (ThT). ThT is a fluorescent probe widely used for the identification of amyloid β‐sheet structure [45, 46]. Samples were prepared by diluting different concentrations of lipopeptide at pH 2, pH 4, or pH 7 (adjusted using HCl and/or NaOH), containing 5 μM of ThT. The excitation wavelength was set to λ_ex_ = 450 nm and spectra were recorded using a Cary Eclipse spectrofluorometer with excitation and emission slits fixed at 5 nm with the temperature maintained at 20°C.

### 2.6 Rheology

Rheological properties were determined using a controlled stress TA Instruments AR‐2000 rheometer. Experiments were performed using a plate–plate geometry (plate radius = 20 cm; gap = 1000 μm). The linear viscoelastic regime was first identified from the dependence of the dynamic storage (G′) and loss (G″) moduli on the oscillatory stress at a fixed frequency ω = 6.283 rad s^−1^. The dependence of G′ and G″ on the oscillatory frequency was then determined at a fixed oscillatory stress σ within the linear regime.

### 2.7 Cell and Tissue Culture

Human dermal fibroblasts from adult donors (HDFa) cells (Sigma‐Aldrich, United Kingdom) were maintained and expanded in Human Fibroblast Expansion Basal Medium (Thermo Fisher Scientific) supplemented with Low Serum Growth Supplement (LSGS), 20 mM HEPES, and 1% GlutaMAX. The cells were maintained at pH 7.4, 37°C, and 5% CO_2_ in T75 cell and tissue culture flasks.

### 2.8 MTT Assays for Cytotoxicity

Cytotoxicity was investigated using 3‐(4,5‐dimethylthiazol‐2‐yl)‐2,5‐diphenyltetrazolium bromide (MTT) assays (Sigma‐Aldrich, United Kingdom). Initially, HDFa cells were seeded at a confluence of 9 × 10^3^ cells in a volume of 100 μL per well into 96‐well plates and incubated for 24 h in normal fibroblast expansion media. On the second day, the media was washed with PBS and HDFa cells were incubated with DMEM/F12 (5% FBS, 1% glutamine, no phenol red) in the presence of different concentrations of C_16_‐KTTKS and C_16_‐RTTRS. After 72 h incubation in the presence of the lipopeptides, the plates were washed with PBS and incubated for 4 h in media without serum or phenol red +0.5 mg/mL MTT at 37°C, protected from the light. The resulting formazan crystals were dissolved using 100 μL of DMSO for 30 min at 37°C, protected from the light. The resulting absorbances were read at 570 nm and the data subsequently analyzed using GraphPad Prism 8 software.

### 2.9 Picrosirius Red Assay for Collagen Detection

To determine the production of collagen, 5 × 10^3^ HDFa cells were seeded into 96 well plates, using DMEM/F12 media and left to attach overnight inside a cell incubator with a 5% CO_2_ atmosphere at 37°C. On the next day, samples consisting of different concentrations of the lipopeptides in DMEM/F12 were added to the plates and incubated for 72 h inside the cell incubator. Controls were incubated only in media without lipopeptide. After the 72 h incubations, the cells were washed 3× with PBS and fixed with ice cold ethanol for at least 20 min inside an ultra‐freezer for collagen fixation. A final incubation with Direct red 80 diluted in picric acid 1% at a concentration of 1 mg/mL was conducted inside a 4°C cold chamber overnight for collagen staining. The plate was washed with ultrapure water to remove any excess of dye and a 100 μL of 1 M NaOH was added to each well to dilute the stained collagen fibers. The resulting absorbance was quantified using an Infinite 50 Tecan (Switzerland) instrument at a fixed wavelength of 492 nm. To calculate the amount of collagen produced, a standard calibration curve made using rat tail collagen (Sigma‐Aldrich) was applied to determine the collagen (mg/mL) of each well. The collagen per cell was calculated using the MTT assay data to estimate cell activity for each concentration of lipopeptide.

## 3 Results and Discussion

**SCHEME 1:**
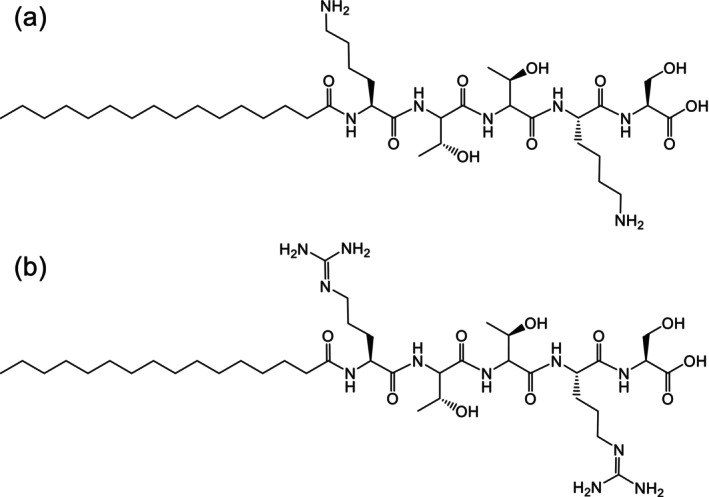
Molecular structures for (a) C_16_‐KTTKS and (b) C_16_‐RTTRS.

We compare the self‐assembly and bioactivity (cytocompatibility and collagen stimulating activity) of C_16_‐KTTKS and C_16_‐RTTRS (Scheme 1), which contain homologous sequences with substitution of cationic residues from lysine to arginine. The self‐assembly of these sequences may be pH‐dependent due to the pH‐dependent charge on the peptide sequences; indeed, C_16_‐KTTKS shows pH‐dependent nanostructures [26]. Here, this is compared with the behavior of the homologue C_16_‐RTTRS and pH‐dependent self‐assembly is examined for both lipopeptides. We also explore the effect of counterions on self‐assembly, comparing the association behavior of acetate and TFA salts, these having very different acid dissociation constants.

Based on our prior work on C_16_‐KTTKS and similar sequences, we expect these lipopeptides to mainly form β‐sheet fibril structures at pH 7 and possibly lower pH. To detect the CAC for fibril formation we used thioflavin (T) fluorescence, a dye that binds to “amyloid” β‐sheet structures [45, 46, 47]. The data are shown in SI Figure S1–S8, and for all conditions, a discontinuity in the ThT fluorescence intensity as a function of concentration enables determination of CAC values. These are tabulated in Table 1. A general trend for a given pH/counterion condition is for C_16_‐RTTRS to have a slightly lower CAC indicating that it is slightly more hydrophobic than C_16_‐KTTKS. The data indicate that both lipopeptides (in both salts) aggregate above a CAC in the range 0.010–0.078 wt%. It is notable that in some cases, the spectra contain small sharp peaks, especially for the acetate salts, and most notably at pH 4. This is reproducible and is consistent with our previously reported ThT fluorescence spectrum for C_16_‐KTTKS (acetate) at pH 4 [21]. A detailed explanation is presently lacking but it may be due to excimer formation within the aggregates. The CAC data show trends in that the values are lower for C_16_‐KTTKS in acetate form than for TFA salts at either pH, whereas for C_16_‐RTTRS the CAC values are lower for the TFA salt than the acetate salt. These effects may be due to the differences in pKa values and steric hindrance, which influence the association of the counterion with the side chain amine or guanidinium groups.

**TABLE 1:** Values of critical aggregation concentration (CAC).

Sample	pH	Salt	CAC (wt%) ± 0.005 wt%
C_16_‐KTTKS	4	TFA	0.078
C_16_‐KTTKS	4	Acetate	0.011
C_16_‐KTTKS	7	TFA	0.073
C_16_‐KTTKS	7	Acetate	0.036
C_16_‐RTTRS	4	TFA	0.010
C_16_‐RTTRS	4	Acetate	0.034
C_16_‐RTTRS	7	TFA	0.014
C_16_‐RTTRS	7	Acetate	0.033

Further studies on lipopeptide conformation and self‐assembled nanostructures were performed at concentrations well above the CAC values, typically for 1 wt% solutions.

**FIGURE 1:**
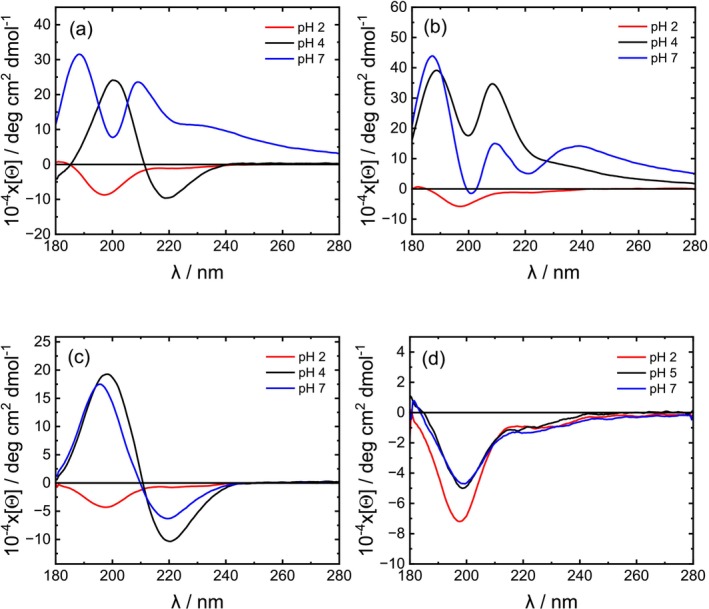
CD spectra for 1 wt% concentration solutions at pH values indicated of (a) C_16_‐KTTKS (TFA), and (b) C_16_‐KTTKS (acetate), (c) C_16_‐RTTRS (TFA), (d) C_16_‐RTTRS (acetate).

The peptide conformation was probed using CD spectroscopy. The spectra for 1 wt% solutions of the two lipopeptides in the two salt forms at pH 2, pH 4/5 or pH 7 are shown in Figure 1. The spectra for C_16_‐KTTKS (TFA) in Figure 1a reveal a transition from unordered structure at pH 2 to β‐sheet structure at pH 4 with a maximum in the spectrum at 200 nm and a minimum around 218 nm [48, 49, 50, 51]. At pH 7, the spectrum shows a minimum at 200 nm and a shoulder minimum near 220 nm with a positive maximum in between. These features are consistent with our previous reports for the TFA salt of this peptide [29], and are ascribed to a β‐sheet conformation with red‐shifted CD spectrum due to light scattering effects [52] in the nanotape structures (discussed below) since the solution is cloudy. Similar effects are observed in the C_16_‐KTTKS (acetate) spectra shown in Figure 1b with a transition from disordered at pH 2 to β‐sheet at higher pH, with red‐shifted spectra with an additional plasmon peak centered at 210 nm, these spectra again being consistent with our previous report for C_16_‐KTTKS (acetate) [25]. The spectra for C_16_‐RTTRS (TFA) in Figure 1c show a transition from disordered at pH 2 to β‐sheet at pH 4 and pH 7. In contrast, the spectra for C_16_‐RTTRS (acetate) in Figure 1d show retention of disordered conformation across the range pH 2–7. This is consistent with the formation of predominant micelles as revealed by cryo‐TEM imaging and SAXS measurements discussed below.

The changes in the CD spectra for both lipopeptide TFA salts and the C_16_‐RTTRS (TFA) salt from disordered structures at pH 2 to β‐sheet at higher pH are consistent with the transition from micelle structures to nanotapes revealed by cryo‐TEM and SAXS as discussed in the following. In comparison, C_16_‐RTTRS (acetate) retains a disordered conformation across the range pH 2–7, which is also consistent with the findings from cryo‐TEM and SAXS of predominant spherical micelle structure in solutions of this lipopeptide salt.

**FIGURE 2:**
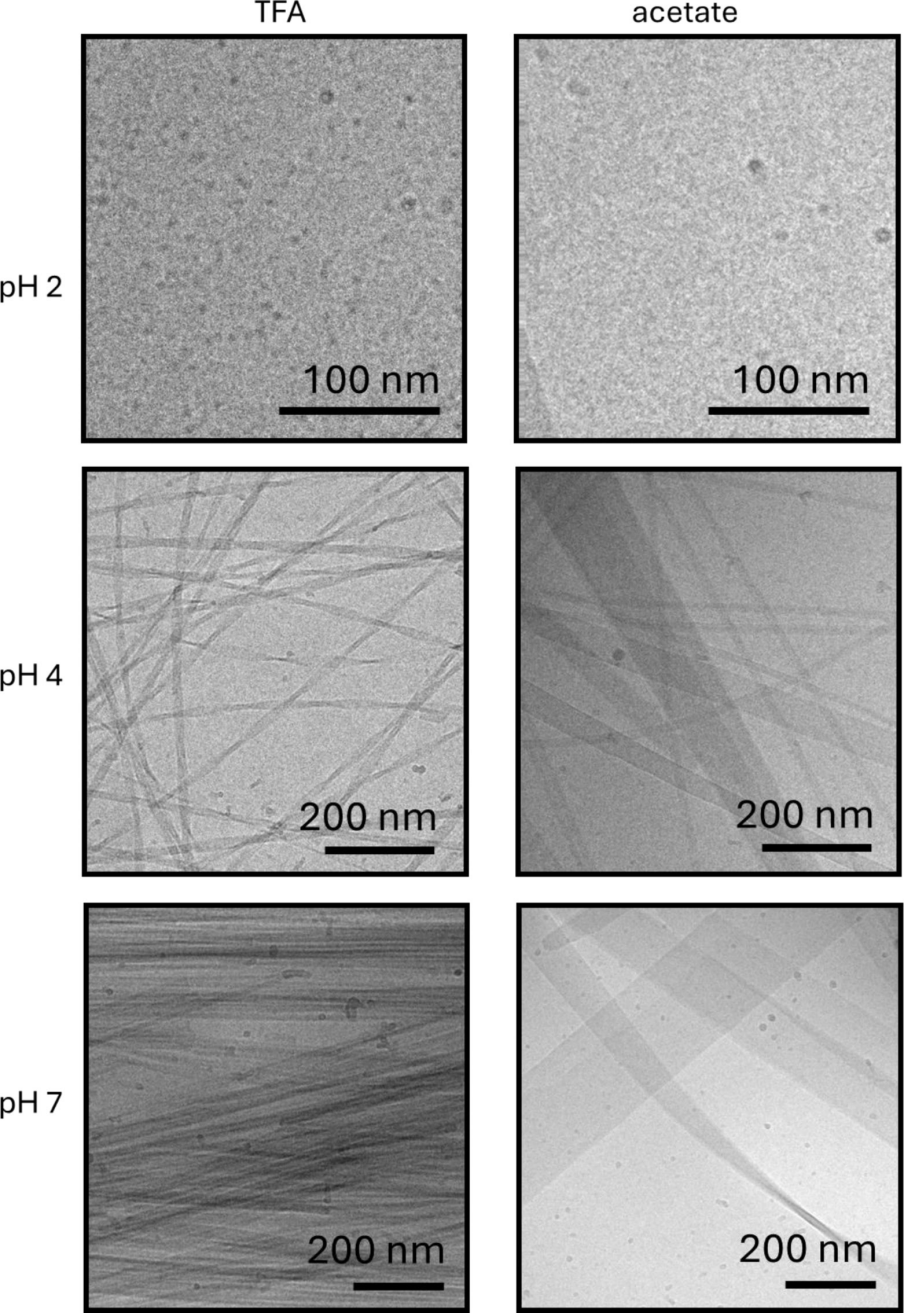
Cryo‐TEM images for 1 wt% solutions of C_16_‐KTTKS at pH values shown and with counterions indicated.

**FIGURE 3:**
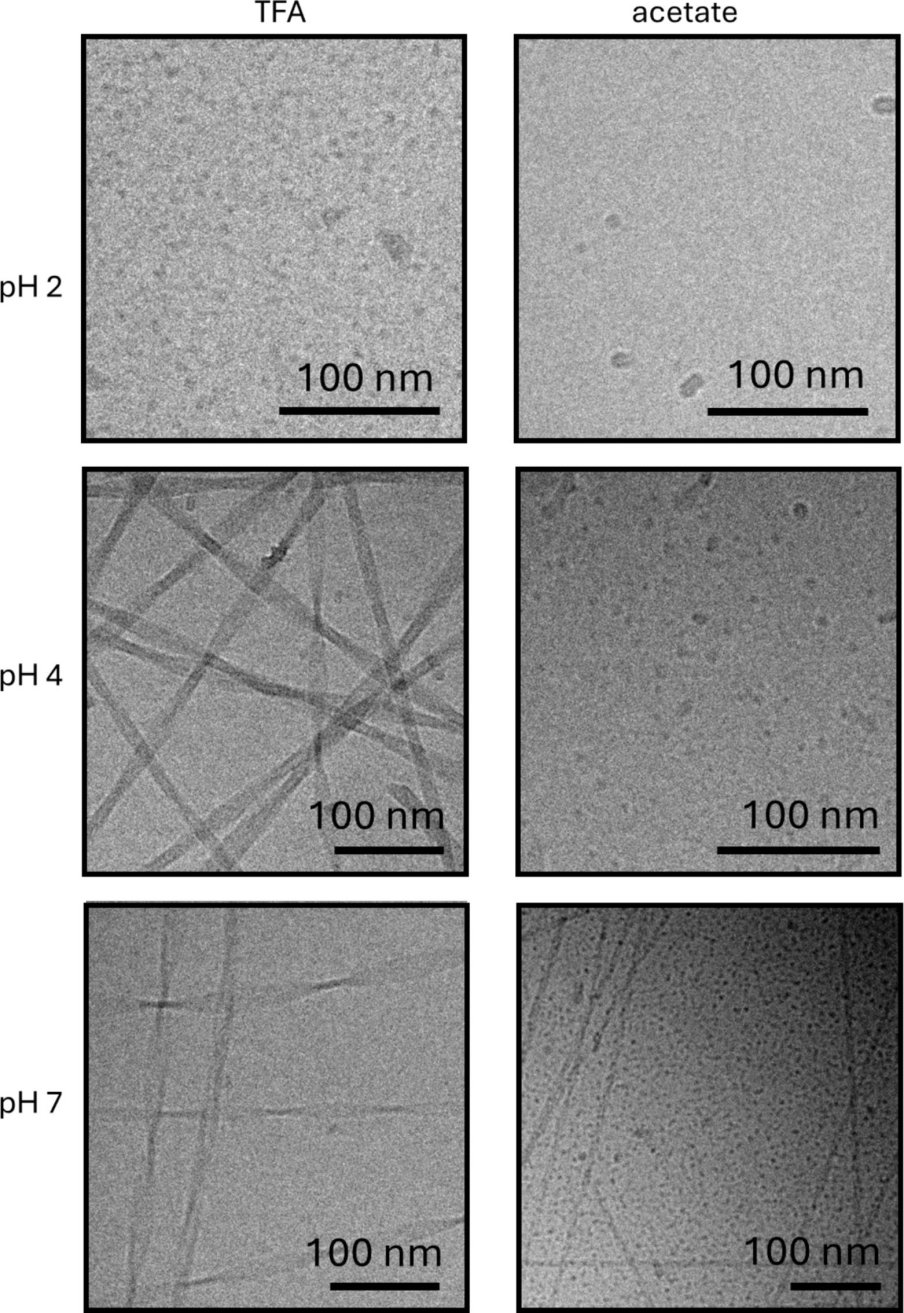
Cryo‐TEM images for 1 wt% solutions of C_16_‐RTTRS at pH values shown and with counterions indicated.

We investigated the nanostructure in aqueous solution of the two lipopeptides as a function of pH in the range pH 2–7 using cryo‐TEM and SAXS. Cryo‐TEM images for C_16_‐KTTKS are presented in Figure 2, and those for C_16_‐RTTRS in Figure 3, with additional images in SI Figures S9–S20. The representative images for C_16_‐KTTKS at pH 2, both TFA and acetate salts, show a predominant morphology of small spherical micelles with a population of extended nanotapes (shown in SI Figures S9 and S10). The population of nanotapes was higher for the acetate salt. These findings are consistent with SAXS discussed below, and the micelle structure is also consistent with the disordered conformation revealed by CD. We previously reported a micelle structure for C_16_‐KTTKS (TFA) at pH 2 on the basis of AFM and SAXS [26]. The cryo‐TEM images in Figure 2 for C_16_‐KTTKS at pH 4 show twisted nanotapes for the TFA salt but wider straighter nanotapes for the acetate salt. At pH 7, extended and straight nanotapes are also observed for either salt, consistent with previous reports [25, 26, 29]. The nanotapes for the TFA salt appear thinner on average and for either salt only occasional twisted tapes were observed, i.e., they appear quite inflexible.

Although C_16_‐KTTKS does not show a difference in overall nanostructure (i.e., nanotapes or micelles) when comparing TFA and acetate salts for a given pH value, a nanostructure change was observed for C_16_‐RTTRS when considering the two salt forms at pH 4 and pH 7 as evident from the cryo‐TEM images in Figure 3. There is a significant population of micelles for the acetate salts at these pH values, which is not observed for the TFA salt, although for the acetate salt the micelles coexist with a lower population of thin twisted nanotapes. The morphology at pH 2 is similar for both salts, being predominantly spherical micelles with a small population of twisted nanotapes. The populations of the nanostructures can be estimated from the weightings of the respective form factors in the SAXS data fitting discussed below.

**FIGURE 4:**
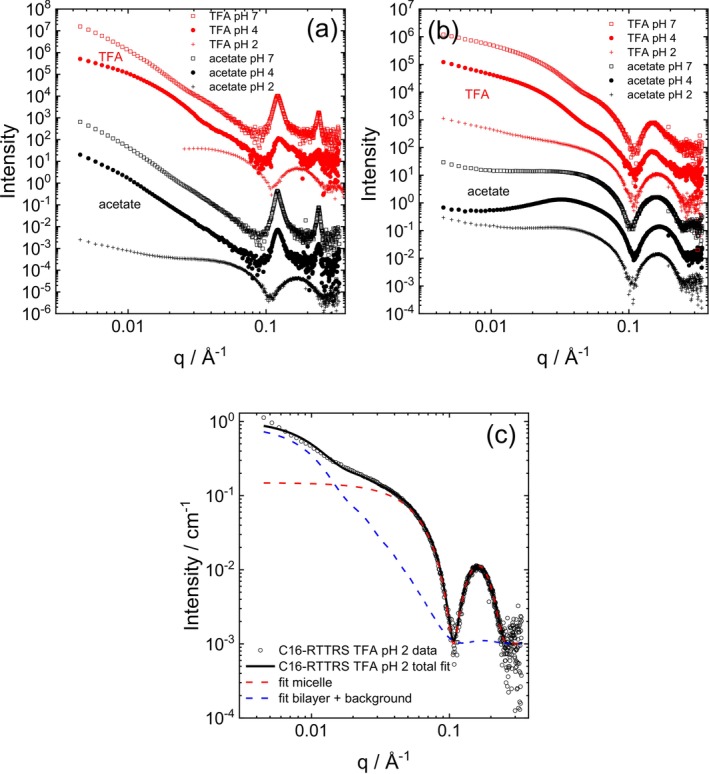
SAXS data at pH values indicated for 1 wt% samples. (a) C_16_‐KTTKS (data for TFA pH 2 is that reported previously, [26] measured with a different setup and *q* range), (b) C_16_‐RTTRS. Data sets are shifted by multiplication for ease of visualization, and only every fifth data point is shown. (c) Representative fit of data accounting for coexisting spherical micelle and nanotape structures, data for C_16_‐RTTRS TFA pH 2 (every fifth data point shown) along with components of the form factor model discussed in the text (fit parameters in SI Table S2).

The nanostructures in aqueous solution for the two lipopeptides at different pH and for both salts were also probed using SAXS, which provides an in situ determination of the sample‐averaged nanostructure. SAXS data is presented in Figure 4, and fitted data is shown in SI Figure 21 for C_16_‐KTTKS and SI Figure 22 for C_16_‐RTTRS. A comparison of the data for C_16_‐KTTKS and C_16_‐RTTRS reveals overall trends in that most intensity profiles (for both salts, at higher pH) for the former lipopeptide (Figure 4a) show a form factor corresponding to multilamellar nanotapes with two orders of Bragg reflection evident at high *q*, corresponding to a layer spacing *d* = 52 Å (SI Table S1). In contrast to the data at higher pH, the SAXS data for C_16_‐KTTKS acetate salt at pH 2 shows distinct features of a spherical micelle form factor, consistent with our previous report for the TFA salt [26]. The SAXS data for C_16_‐RTTRS in Figure 4b shows distinct features to that for C_16_‐KTTKS under comparable pH/salt conditions. For the acetate salt at the higher pH values, the SAXS does not show Bragg peaks, i.e., C_16_‐RTTRS does not self‐assemble into nanotapes comprising large numbers of bilayers. Instead, under these conditions the form factor shows features of tapes built from only a small number of lipopeptide bilayers (*N* = 5 from data fitting, SI Table S2). In contrast, C_16_‐RTTRS (TFA) at pH 2 and C_16_‐RTTRS (acetate) at all pH values show micelle form factor features at high *q*, with only a small contribution from fibrils (a population of which is evident in the cryo‐TEM images in Figure 2) leading to a non‐zero intensity slope at low *q*. This low *q* intensity slope is more apparent for the pH 2 TFA solution and the higher pH acetate solution. The separate contributions from the two self‐assembled structures are highlighted in a representative fit of data for C_16_‐RTTRS (TFA) at pH 2 shown in Figure 4c. The respective weightings of the form factor components, which provide a measure of the populations of the species, are listed in SI Table S2. The contribution of micelles predominates giving the form factor shape at high *q*, but the population of tapes apparent in the cryo‐TEM images gives rise to enhanced scattering at low *q*. The SAXS data for C_16_‐RTTRS reveals both pH‐dependence and, at pH 4 and pH 7, a dependence on the nature of the counterion. SAXS also reveals that the self‐assembly of this lipopeptide is distinct from that of C_16_‐KTTKS, which shows less dependence on counterion type, comparing acetate and TFA salts, and which also forms multilamellar nanotapes at high pH in contrast to C_16_‐RTTRS under these conditions. It should be noted that the CAC values from ThT assays for C_16_‐RTTRS (acetate) reported in Table 1 are presumably sensitive to the minor component of β‐sheet nanotapes revealed by cryo‐TEM and SAXS data analysis.

SAXS data at pH 2 and pH 7 were fitted in a manner consistent with the shape of the intensity profiles as qualitatively discussed above, and the nanostructures present in the cryo‐TEM images. For C_16_‐KTTKS, the data can be fitted using a Gaussian bilayer form factor [31, 53] along with a lamellar structure factor, calculated using the Caillé model for fluctuating lamellae [54]. The fitted data is shown in SI Figure 21 and SI Figure 22 and the fit parameters are listed in SI Table S1 and SI Table S2. For C_16_‐KTTKS, at high *q* the structure factor predominates and sharp Bragg peaks are observed in the SAXS data with spacing 52 Å at pH 7 and pH 4. This value is in good agreement with our previously reported value for C_16_‐KTTKS (acetate) [25]. At pH 2, the SAXS data indicates that C_16_‐KTTKS (acetate) forms spherical micelles and the data was well fitted with a core‐shell sphere structure factor (SI Figure 21). We reported that C_16_‐KTTKS (TFA) forms micelles at pH 2 and the previous data [26] have been refitted and the parameters are in good agreement with those for the C_16_‐KTTKS (acetate) pH 2 data (SI Table S1).

Since cryo‐TEM and qualitative analysis of SAXS profiles indicates the presence of coexisting structures for C_16_‐RTTRS for most pH values for either salt, we used a model comprising the form factor of a core‐shell sphere to describe a micelle along with a bilayer represented by a sum of three Gaussian functions [53, 55, 56] to describe the electron density profile across the lipopeptide bilayer (plus a flat background term). As shown by the example data for C_16_‐RTTRS (TFA) at pH 2 in Figure 4c, this physically motivated model describes the data very well. The separate components of the fit are shown in Figure 4c, which illustrates that the high *q* form factor oscillation is due to the micellar structure, while the contribution from the bilayer affects only the slope of the intensity at low *q*. The corresponding fit parameters for the C_16_‐RTTRS data are listed in SI Table S2 and indicate for the micelle an outer radius *R*_o_ = 34 Å and inner radius *R*_i_ = 13 Å. These are reasonable considering that the inner core comprises C_16_ chains with conformational disorder and the outer part comprises pentapeptides with estimated length in extended conformation of around 20 Å. For the bilayer, the thickness *t* = 30 Å is consistent with an interdigitated structure of lipopeptide molecules.

For C_16_‐RTTRS, the SAXS data at pH 2 (Figure 4b) show differences from a bilayer nanotape form factor, with a lower intensity slope at low q and a broader structure factor maximum at high q. The data were fitted with a combination of the form factor of predominant spherical micelles represented as core‐shell spheres, along with a smaller contribution from the form factor of bilayer‐based tapes (Gaussian bilayer model). This model, based on the cryo‐TEM images, provides an excellent description of the data. The fit parameters are listed in SI Table S2. The outer radius is (34.0 ± 2.0) Å with a core radius 12.4 Å. These parameters are reasonable for a core comprising a C_16_ alkyl chain and a pentapeptide corona. The thickness of the bilayer tape is 28‐30 Å which corresponds to a bilayer of interdigitated molecules. At pH 4, the data for C_16_‐RTTRS (TFA) resemble the SAXS profiles for C_16_‐KTTKS (TFA) at the corresponding pH values and this results from population of nanotape bilayer structures. However, the form factor maximum at higher *q* is broader for C_16_‐RTTRS and this indicates the presence of some micelles and a reduction in multilamellar order. At pH 7, the data can be fitted with a form factor for nanotapes described using the Gaussian bilayer model. The sharpness of the peak centered at *q* = 0.138 Å^−1^ requires consideration of structure factor (which also slightly increases the low *q* slope of the intensity profile), which was described using a model for a lamellar structure with *N* = 5 repeats of a lamellar spacing *d* = 42 Å (the other fit parameters are listed in SI Table S2). Thus, C_16_‐RTTRS shows notable differences in pH‐dependent SAXS data compared with C_16_‐KTTKS and consistent with cryo‐TEM, there is evidence for a substantial population of micelles at pH 2, and some micelles at pH 4 whereas at pH 7 the system contains some nanotapes. These have less multi‐layer repeats than for C_16_‐KTTKS and this in turn may lead to greater flexibility of β‐sheets leading to the observed greater twisting observed in cryo‐TEM for the C_16_‐RTTRS nanotapes compared with C_16_‐KTTKS.

**FIGURE 5:**
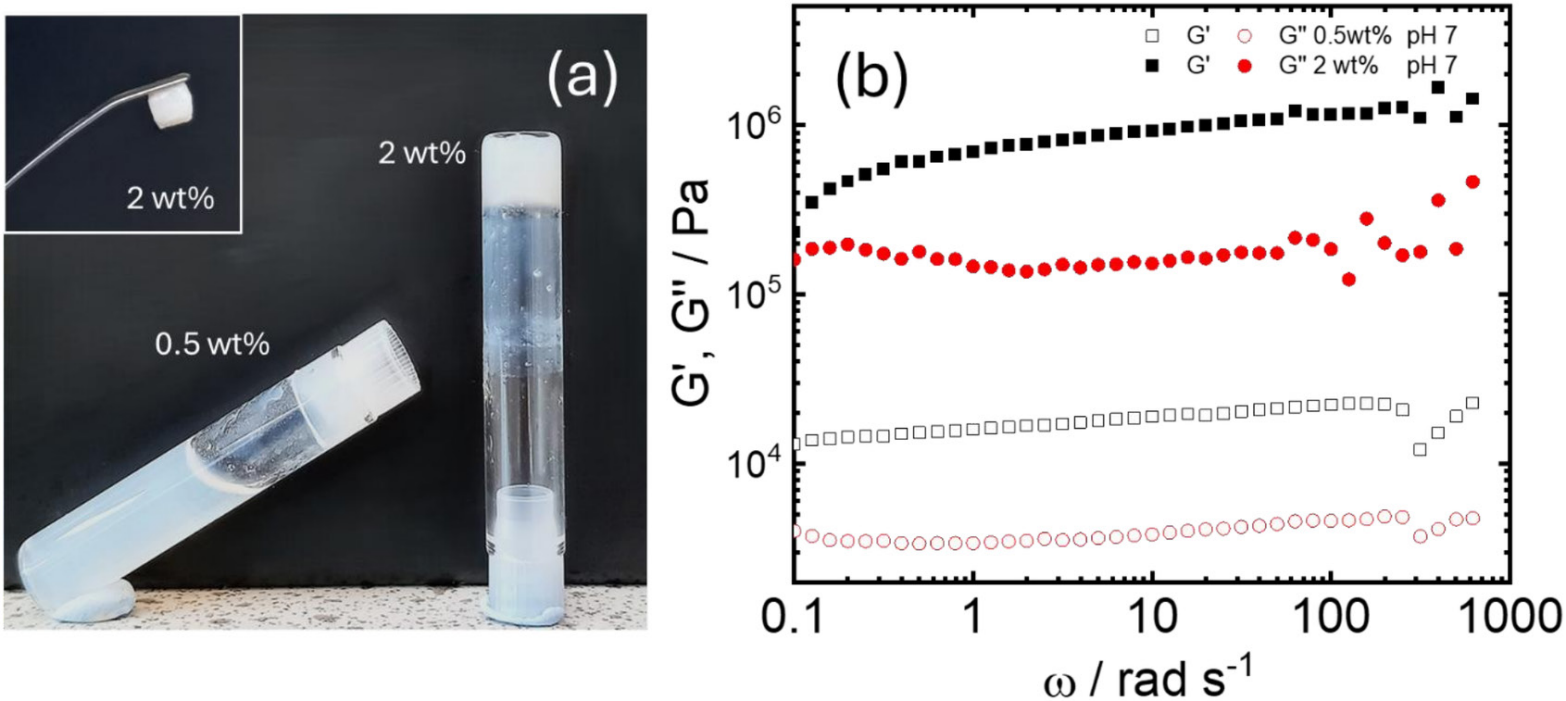
(a) Tube inversion showing hydrogel formation of C_16_‐KTTKS (TFA) at 0.5 wt% (soft gel) and 2 wt% (self‐supporting gel). (b) Frequency dependence of dynamic shear moduli for gels containing 0.5 wt% or 2 wt% C_16_‐KTTKS (TFA) measured at shear stress σ = 100 Pa and σ = 200 Pa, respectively.

We noted that C_16_‐KTTKS forms cloudy hydrogels at pH 7 at concentrations above 0.5 wt% for either TFA or acetate salts. This was not noted for C_16_‐RTTRS under these conditions. Figure 5a shows a soft gel for an 0.5 wt% sample, unable to resist flow upon tube inversion; however, a self‐standing gel was observed for a 2 wt% C_16_‐KTTKS sample. The inset in Figure 5a shows that the 2‐wt% C_16_‐KTTKS makes a relatively robust gel, which can be molded into a cube, which does not fall under its own weight on the tip of an inverted spatula. Differently from the gel made at 0.5 wt.%, the 2 wt.% hydrogel does not flow upon tube inversion. The mechanical and viscoelastic properties of a hydrogel strongly impact how it interacts with cells, with the hardness of a hydrogel playing an important role in cell anchorage, viability, and proliferation [57, 58]. The viscoelastic properties of gels in Figure 5a were studied by dynamic shear rheology. An oscillatory stress sweep experiment was run to identify the linear regime for 0.5 and 2 wt% C_16_‐KTTKS gels (SI Figure S23). The stress sweep curves were used to choose a fixed shear stress for frequency sweep measurements σ = 100 Pa for a 0.5 wt% C_16_‐KTTKS gel or σ = 200 Pa for a 2 wt% C_16_‐KTTKS gel. Figure 5b shows the frequency sweep data measured for the gels in Figure 5a. As expected, G′ and G″ are higher for the self‐standing 2 wt.% gel than for the 0.5 wt% soft gel. In addition, the moduli are weakly dependent on frequency at high frequency, again consistent with a gel structure. These gels may have future applications in 3D cell culture for tissue engineering or for slow release of encapsulated cargo, for drug release for example.

**FIGURE 6:**
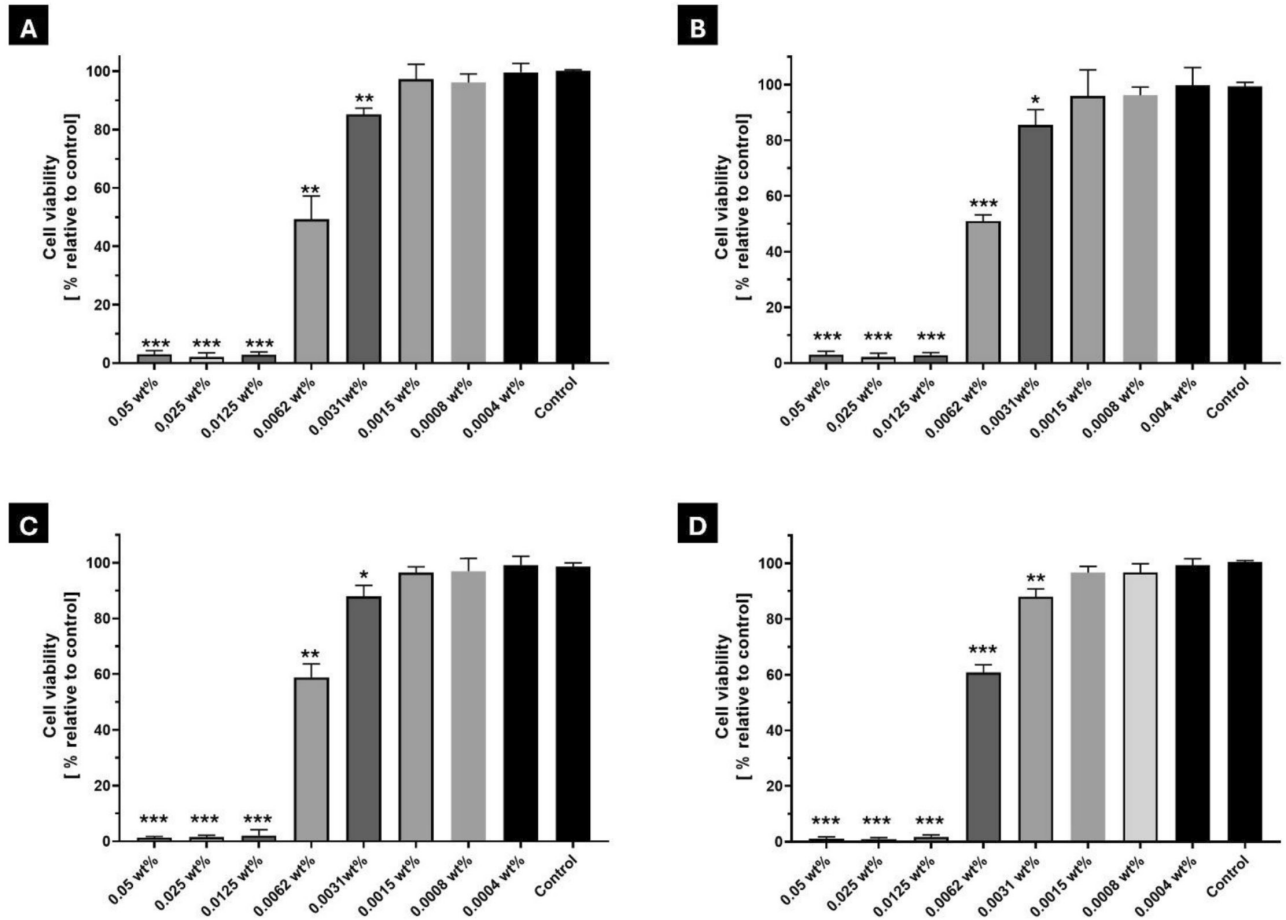
MTT assays for (A) C_16_‐KTTKS TFA, (B) C_16_‐KTTKS acetate, (C) C_16_‐RTTRS TFA, (D) C_16_‐RTTRS acetate. The statistical test used for data treatment was ANOVA, *n* = 3 with Bonferroni correction for multiple comparisons. ** *p* ≤ 0.01.

We examined the effect of the lipopeptides in different salt forms on bioactivity through assays of cell viability and collagen production studies. Assays of cell viability using MTT showed little difference between the TFA and acetate salts for C_16_‐KTTKS (Figure 6A,B, respectively), as both forms caused a significant decrease in cell viability starting at 0.0062 wt%, with approximately 50% less cell viability compared with control cells incubated only in DMEM. At concentrations of 0.0125 wt% and above, cell viability was negligible. The MTT assays reveal that C_16_‐RTTRS was better tolerated, with both salts causing a decrease of approximately 40% in cell viability at 0.0062 wt% (Figure 6C,D). Little difference was observed between the TFA and acetate salts of C_16_‐RTTRS, although the acetate form showed marginally improved cytocompatibility (Figure 6D). These findings suggest that, at the concentrations evaluated, the observed cytotoxicity is more likely driven by the cationic nature of the lysine/arginine residues and interactions with the lipid portion of the lipopeptide than by the presence of TFA at levels sufficient to induce a counterion‐specific toxic effect [18, 59, 60].

**FIGURE 7:**
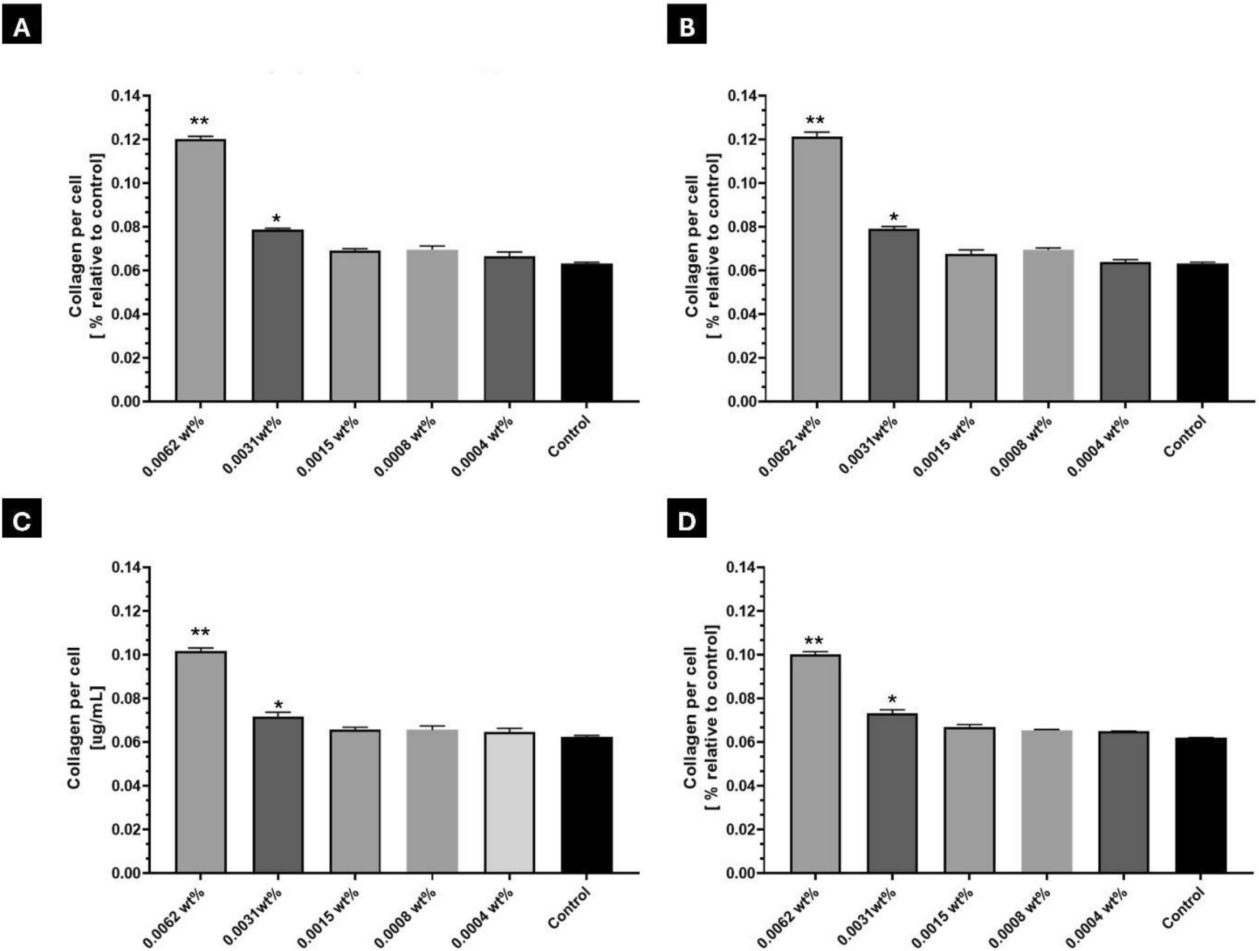
Collagen per cell quantification determined by picrosirius red assay for (A) C_16_‐KTTKS TFA, (B) C_16_‐KTTKS acetate, (C) C_16_‐RTTRS TFA, (D) C_16_‐RTTRS acetate. The statistical test used for data treatment was Kruskal–Wallis with Dunn's correction for multiple comparisons, *n* = 3. *p ≤ 0.05, **p ≤ 0.01.

We quantified collagen production using picrosirius red staining, a technique that can be used to identify and quantify Type I and Type III collagen [61], and which we have previously applied to evaluate collagen production induced by C_16_‐KTTKS [21]. Total collagen production by the cells is shown in Figure S24. Data showing collagen production normalized per cell is shown in Figure 7, using cell viability data obtained from the MTT assays as the basis for the calculations. We observed a statistically significant increase in collagen production in cells incubated with 0.0031 and 0.0062 wt% for either lipopeptide in either salt form. The collagen production (and collagen per cell) is slightly higher for C_16_‐KTTKS (either salt) than for C_16_‐RTTRS. Because of the high cytotoxicity observed using higher concentrations, groups incubated with 0.0125 to 0.05 wt% lipopeptide were not included in the collagen per cell analysis, although significant increases in total collagen production were still detected in these groups (Figure S24). Again, no differences in collagen production per cell were observed between the TFA and acetate salts of C_16_‐KTTKS or C_16_‐RTTRS. The total collagen production for C_16_‐KTTKS (acetate) is of similar magnitude to that previously reported [21]; however, the concentration range where we observe a significant increase in collagen production, especially 0.05 wt%, is higher than the previously reported range where a substantial increase was observed at 0.008 wt% [21], and this in turn leads to differences in collagen per cell. This may reflect differences in protocols, and most especially in the human dermal fibroblast origins.

## 4 Conclusions

SAXS together with cryo‐TEM reveal significant differences in the self‐assembly of C_16_‐RTTRS compared with C_16_‐KTTKS. Both lipopeptides are studied well below the respective pKa values for lysine (pH 10.5) or arginine (pH 12.5) side chains and so the peptides should be fully charged (both +1 considering the charge on the C terminus). C_16_‐KTTKS forms predominantly micelles at pH 2 and nanotapes at higher pH, and there is little effect of counterion on these qualitative findings, although the nanotape morphology does appear to be influenced. Examining the cryo‐TEM images, the nanotapes are wider and appear more flexible for the acetate salt. This may reflect the strength of counterion binding to the peptide sequence within the nanotapes. There are distinctive features in the self‐assembly of C_16_‐RTTRS in comparison with C_16_‐KTTKS, in particular, the acetate form of C_16_‐RTTRS shows micelle structures across a wide pH range, not just at pH 2. C_16_‐KTTKS shows more pH‐stable nanotape structures and is less perturbed by the nature of the salt. In addition, the SAXS data shows that nanotapes formed by the TFA salt of C_16_‐RTTRS do not have the well‐defined multilamellar order of those of C_16_‐KTTKS, and they appear more flexible (more twisted) in cryo‐TEM images. The differences in lamellar ordering are ascribed to differences in electrostatic interactions for the two lipopeptides and in hydrogen bonding within the lamellae, as well as longer‐range lamellar fluctuations, which may influence inter‐lamellar ordering. It appears that C_16_‐KTTKS has a more cohesive structure than C_16_‐RTTRS, which may also influence the dynamics of molecular motion [62], although in the case of these two lipopeptides, it has little effect on the bioactivity in our cytocompatibility and collagen assays. Differences in the molecular packing, hydrogen bonding, and dynamics are the subject of ongoing studies in our group, including MD simulations.

The fluorescence ThT assays indicate that both C_16_‐KTTKS and C_16_‐RTTRS self‐assemble at low concentrations, with the notable effect that substitution of lysine residues for arginine residues lowered the CAC across the pH range examined. The type of counterion also affected the CAC. Consistent with the cryo‐TEM and SAXS studies of self‐assembly, the CD data confirm pH‐dependent changes in secondary structure with disordered conformation for lipopeptides at pH 2 and C_16_‐RTTRS with acetate counterions for pH 2–7, consistent with micelle formation. The other samples (C_16_‐KTTKS at pH 4 or 7 with either counterion, C_16_‐RTTRS with TFA at pH 4 or 7) show β‐sheet signatures in CD, consistent with the formation of nanotape structures. The pH‐dependent self‐assembly and conformation opens the door for the use of these peptides as pH responsible systems of drug delivery, controlling the dissociation or aggregation of the sequences by pH. This is interesting considering the potential of the peptides in injury healing, since injury and tissues under inflammatory process often present lower pH compared with healthy tissue. Our identification of conditions for hydrogel formation of C_16_‐KTTKS at pH 7 may also be useful for cell culture and tissue engineering as well as slow‐release applications, with pH‐dependent release also possible. It is notable that C_16_‐RTTRS does not form hydrogels at the same conditions, and the difference may reflect the greater rigidity and/or bundling of nanotapes observed in cryo‐TEM images for C_16_‐KTTKS.

Both lipopeptides (in either salt form) showed similar cytocompatibility profiles across the concentration range examined. Cytotoxicity was observed for concentrations 0.0062 wt% and above, this lying below the CAC values established by ThT fluorescence assays (Table 1). This indicates that unaggregated peptide influences cell viability in a dose‐dependent manner. Both lipopeptides stimulate collagen production by human dermal fibroblasts, especially at 0.0062 wt% (considering collagen production per cell), and this is hardly affected by the peptide sequence or the counterion, although the activity is somewhat higher for C_16_‐KTTKS with an approximate doubling of collagen per cell compared with control (media only).

In summary, sequence variation, pH, and counterion selection can be used to control the self‐assembly of C_16_‐KTTKS derivatives, and it is possible to form hydrogels under defined conditions. The compounds are cytocompatible with human dermal fibroblasts over a range of concentrations and, at defined concentrations, show significant collagen stimulation activity. The bioactivity is little affected comparing C_16_‐KTTKS and C_16_‐RTTRS and is not adversely affected by the use of TFA compared to acetate.

## Funding

This work was supported by the Engineering and Physical Sciences Research Council, EP/V053396/1.

## Conflicts of Interest

The authors declare no conflicts of interest.

## Supporting information


**Figure S1:** CAC from ThT fluorescence experiment for C_16_‐KTTKS acetate salt, pH = 4. (A) CAC from fluorescence intensity at λ = 475 and (B) original spectra at lipopeptide concentrations indicated.
**Figure S2:** CAC from ThT fluorescence experiment for C_16_‐KTTKS TFA salt, pH = 4. (A) CAC from fluorescence intensity at λ = 475 and (B) original spectra at lipopeptide concentrations indicated.
**Figure S3:** CAC from ThT fluorescence experiment for C_16_‐KTTKS acetate salt, pH = 7. (A) CAC from fluorescence intensity at λ = 475 and (B) original spectra at lipopeptide concentrations indicated.
**Figure S4:** CAC from ThT fluorescence experiment for C_16_‐KTTKS TFA salt, pH = 7. (A) CAC from fluorescence intensity at λ = 475 and (B) original spectra at lipopeptide concentrations indicated.
**Figure S5:** CAC from ThT fluorescence experiment for C_16_‐RTTRS acetate salt, pH = 4. (A) CAC from fluorescence intensity at λ = 475 and (B) original spectra at lipopeptide concentrations indicated.
**Figure S6:** CAC from ThT fluorescence experiment for C_16_‐RTTRS TFA salt, pH = 4. (A) CAC from fluorescence intensity at λ = 475 and (B) original spectra at lipopeptide concentrations indicated.
**Figure S7:** CAC from ThT fluorescence experiment for C_16_‐RTTRS acetate salt, pH = 7. (A) CAC from fluorescence intensity at λ = 475 and (B) original spectra at lipopeptide concentrations indicated.
**Figure S8:** CAC from ThT fluorescence experiment for C_16_‐RTTRS TFA salt, pH = 7. (A) CAC from fluorescence intensity at λ = 475 and (B) original spectra at lipopeptide concentrations indicated.
**Figure S9:** Additional cryo‐TEM image for 1 wt% solution of C_16_‐KTTKS (TFA) at pH 2.
**Figure S10:** Additional cryo‐TEM image for 1 wt% solution of C_16_‐KTTKS (acetate) at pH 2.
**Figure S11:** Additional cryo‐TEM image for 1 wt% solution of C_16_‐KTTKS (TFA) at pH 4.
**Figure S12:** Additional cryo‐TEM image for 1 wt% solution of C_16_‐KTTKS (acetate) at pH 4.
**Figure S13:** Additional cryo‐TEM image for 1 wt% solution of C_16_‐KTTKS (TFA) at pH 7.
**Figure S14:** Additional cryo‐TEM image for 1 wt% solution of C_16_‐KTTKS (acetate) at pH 7.
**Figure S15:** Additional cryo‐TEM image for 1 wt% solution of C_16_‐RTTRS (TFA) at pH 2.
**Figure S16:** Additional cryo‐TEM image for 1 wt% solution of C_16_‐RTTRS (acetate) at pH 2.
**Figure S17:** Additional cryo‐TEM image for 1 wt% solution of C_16_‐RTTRS (TFA) at pH 5.
**Figure S18:** Additional cryo‐TEM image for 1 wt% solution of C_16_‐RTTRS (acetate) at pH 5.
**Figure S19:** Additional cryo‐TEM image for 1 wt% solution of C_16_‐RTTRS (TFA) at pH 7.
**Figure S20:** Additional cryo‐TEM image for 1 wt% solution of C_16_‐RTTRS (acetate) at pH 7.
**Figure S21:** SAXS data (open symbols) for 1 wt% solutions of C_16_‐KTTKS at pH 2 or pH 7 with acetate or TFA along with model form factor fits (solid lines) described in the text. Fit parameters listed in SI Table S1.
**Figure S22:** SAXS data (open symbols) for 1 wt% solutions of C_16_‐RTTRS at pH 2 or pH 7 with acetate or TFA along with model form factor fits (solid lines) described in the text. Fit parameters listed in SI Table S1.
**Figure S23:** Dynamic shear moduli stress sweep curves generated from a 0.5 (hollow dots and squares) and another 2 wt% C_16_‐KTTKS (squares and dots) hydrogels at a fixed frequency ω = 6.283 rad s^−1^.
**Figure S24:** Total collagen production determined by the picrosirius red assay for (A) TFA salts of C_16−_KTTKS, (B) Acetate salts of C_16−_KTTKS, (C) TFA salts of C_16−_RTTRS and (D) Acetate salts of C_16−_RTTRS. ANOVA with Bonferroni correction for multiple comparisons, *n* = 3.
**Table S1:** Parameters extracted from the fitting of the SAXS data for 1 wt% solutions of C_16_‐KTTKS at pH values indicated. ^a^
**Table S2:** Parameters extracted from the fitting of the SAXS data for 1 wt% solutions of C_16_‐RTTRS at pH values indicated. ^a^

## Data Availability

The data that support the findings of this study are available on request from the corresponding author. The data are not publicly available due to privacy or ethical restrictions.
