# Supplementary material for: Nanostructure and Collagen‐Stimulating Activity of Cationic Pentapeptide Lipopeptides
Source: J Pept Sci. 2026 Jun 19;32(8):e70111. doi: 10.1002/psc.70111 (PMC13280649; doi:10.1002/psc.70111)
Supplement: Supplementary file 1 — Figure S1: CAC from ThT fluorescence experiment for C16‐KTTKS acetate salt, pH = 4. (A) CAC from fluorescence intensity at λ = 475 and (B) original spectra at lipopeptide concentrations indicated. Figure S2: CAC from ThT fluorescence experiment for C16‐KTTKS TFA salt, pH = 4. (A) CAC from fluorescence intensity at λ = 475 and (B) original spectra at lipopeptide concentrations indicated. Figure S3: CAC from ThT fluorescence experiment for C16‐KTTKS acetate salt, pH = 7. (A) CAC from fluorescence intensity at λ = 475 and (B) original spectra at lipopeptide concentrations indicated. Figure S4: CAC from ThT fluorescence experiment for C16‐KTTKS TFA salt, pH = 7. (A) CAC from fluorescence intensity at λ = 475 and (B) original spectra at lipopeptide concentrations indicated. Figure S5: CAC from ThT fluorescence experiment for C16‐RTTRS acetate salt, pH = 4. (A) CAC from fluorescence intensity at λ = 475 and (B) original spectra at lipopeptide concentrations indicated. Figure S6: CAC from ThT fluorescence experiment for C16‐RTTRS TFA salt, pH = 4. (A) CAC from fluorescence intensity at λ = 475 and (B) original spectra at lipopeptide concentrations indicated. Figure S7: CAC from ThT fluorescence experiment for C16‐RTTRS acetate salt, pH = 7. (A) CAC from fluorescence intensity at λ = 475 and (B) original spectra at lipopeptide concentrations indicated. Figure S8: CAC from ThT fluorescence experiment for C16‐RTTRS TFA salt, pH = 7. (A) CAC from fluorescence intensity at λ = 475 and (B) original spectra at lipopeptide concentrations indicated. Figure S9: Additional cryo‐TEM image for 1 wt% solution of C16‐KTTKS (TFA) at pH 2. Figure S10: Additional cryo‐TEM image for 1 wt% solution of C16‐KTTKS (acetate) at pH 2. Figure S11: Additional cryo‐TEM image for 1 wt% solution of C16‐KTTKS (TFA) at pH 4. Figure S12: Additional cryo‐TEM image for 1 wt% solution of C16‐KTTKS (acetate) at pH 4. Figure S13: Additional cryo‐TEM image for 1 wt% solution of C16‐KTTKS (TFA) at pH 7. Figure S [file PSC-32-e70111-s001.docx]

**Supporting Information**

**Nanostructure and Collagen-Stimulating Activity of Cationic Pentapeptide Lipopeptides**

Lucas R de Mello,^1^ Jani Seitsonen,^2^ Valeria Castelletto,^1^ Ian W. Hamley,^1,*^

*^1^ School of Chemistry, Food Biosciences and Pharmacy, University of Reading, Whiteknights, Reading RG6 6AD, U.K.*

*^2^ Nanomicroscopy Center, Aalto University, Puumiehenkuja 2, FIN-02150 Espoo, Finland*


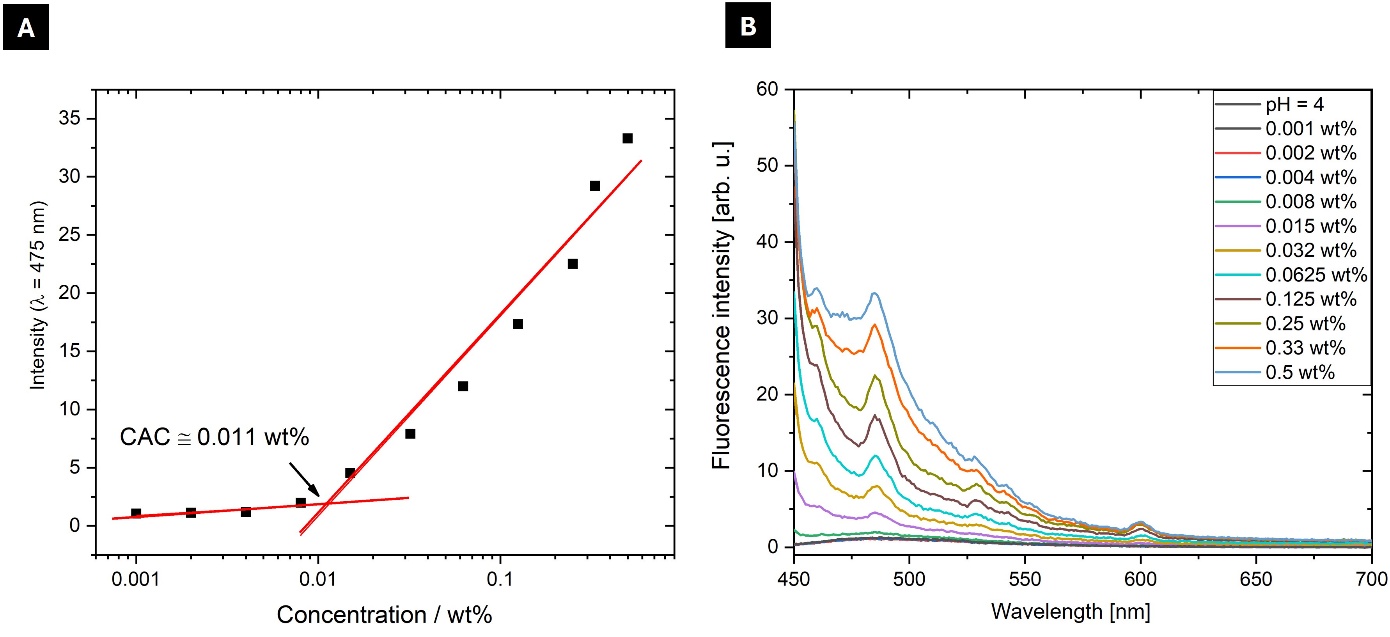


**SI Figure S1**. CAC from ThT fluorescence experiment for C_16_-KTTKS acetate salt, pH = 4. (A) CAC from fluorescence intensity at λ = 475 and (B) original spectra at lipopeptide concentrations indicated.


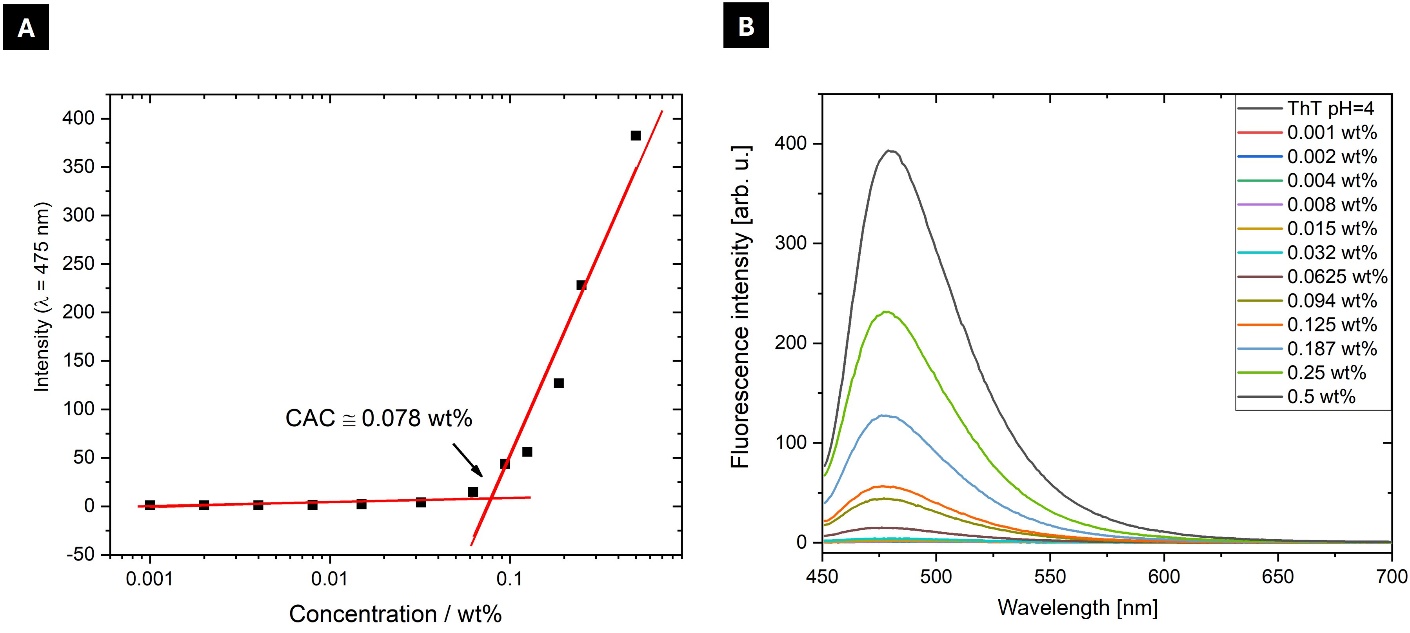
 **SI Figure S2**. CAC from ThT fluorescence experiment for C_16_-KTTKS TFA salt, pH = 4. (A) CAC from fluorescence intensity at λ = 475 and (B) original spectra at lipopeptide concentrations indicated.


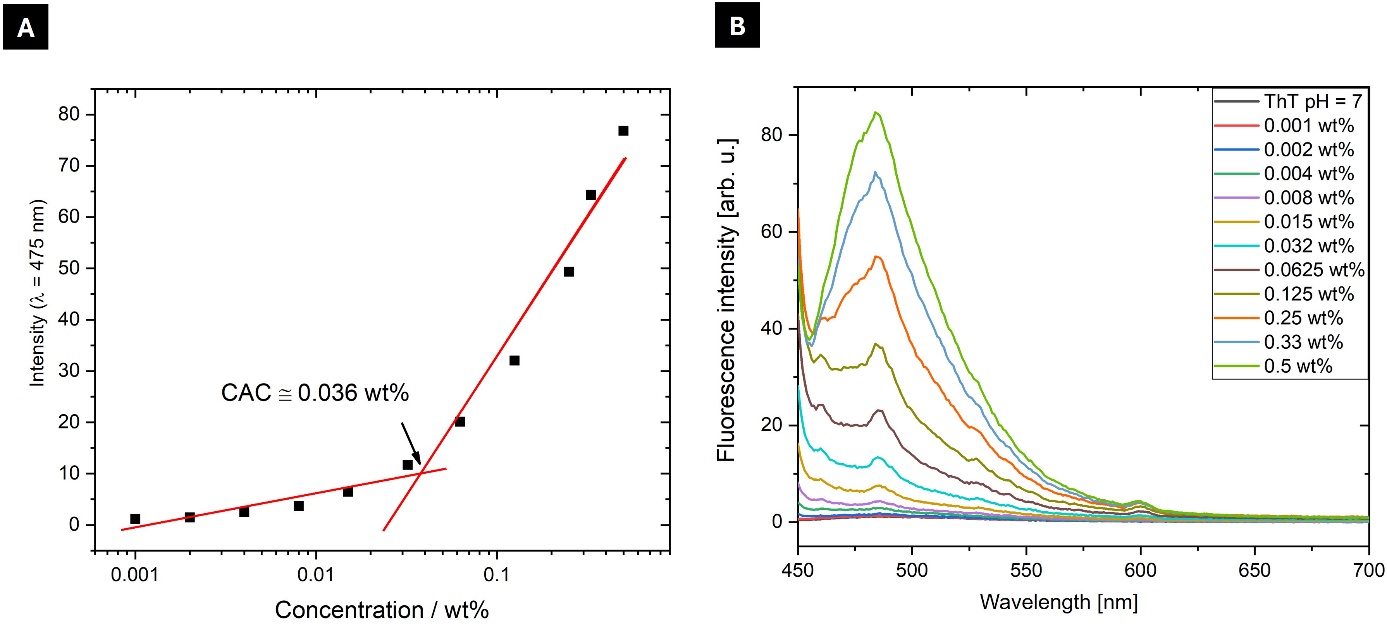


**SI Figure S3**. CAC from ThT fluorescence experiment for C_16_-KTTKS acetate salt, pH = 7. (A) CAC from fluorescence intensity at λ = 475 and (B) original spectra at lipopeptide concentrations indicated.


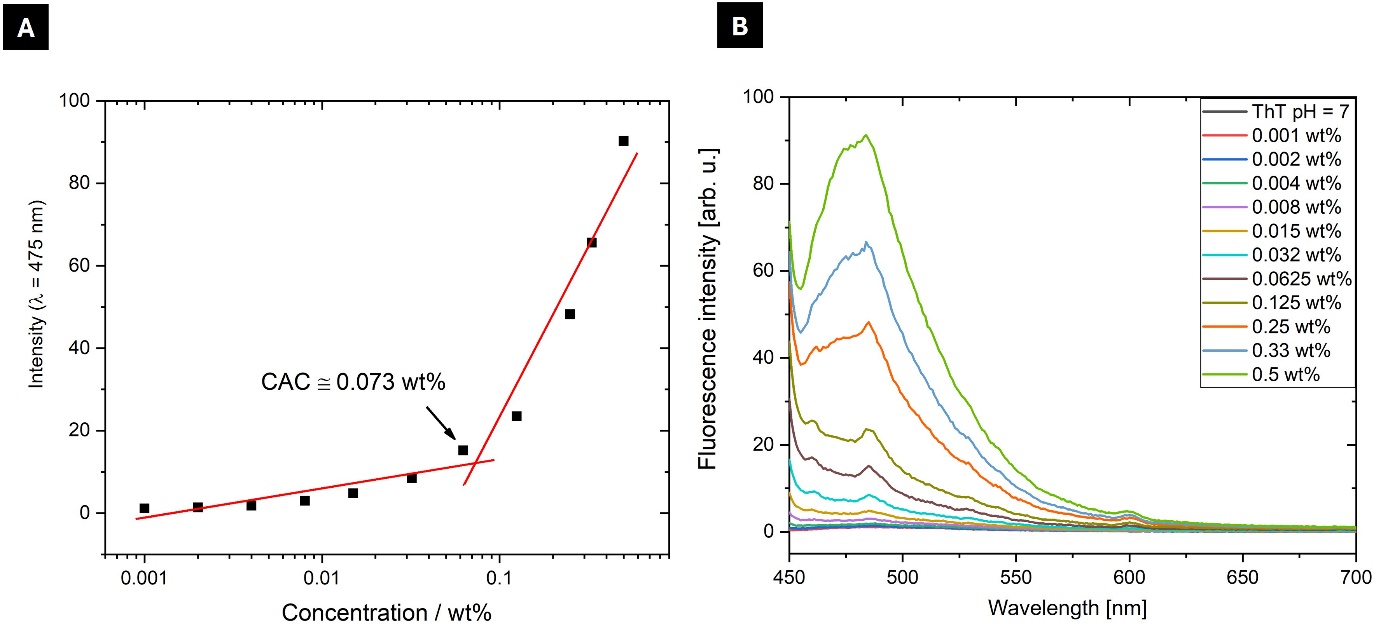


**SI Figure S4**. CAC from ThT fluorescence experiment for C_16_-KTTKS TFA salt, pH = 7. (A) CAC from fluorescence intensity at λ = 475 and (B) original spectra at lipopeptide concentrations indicated.


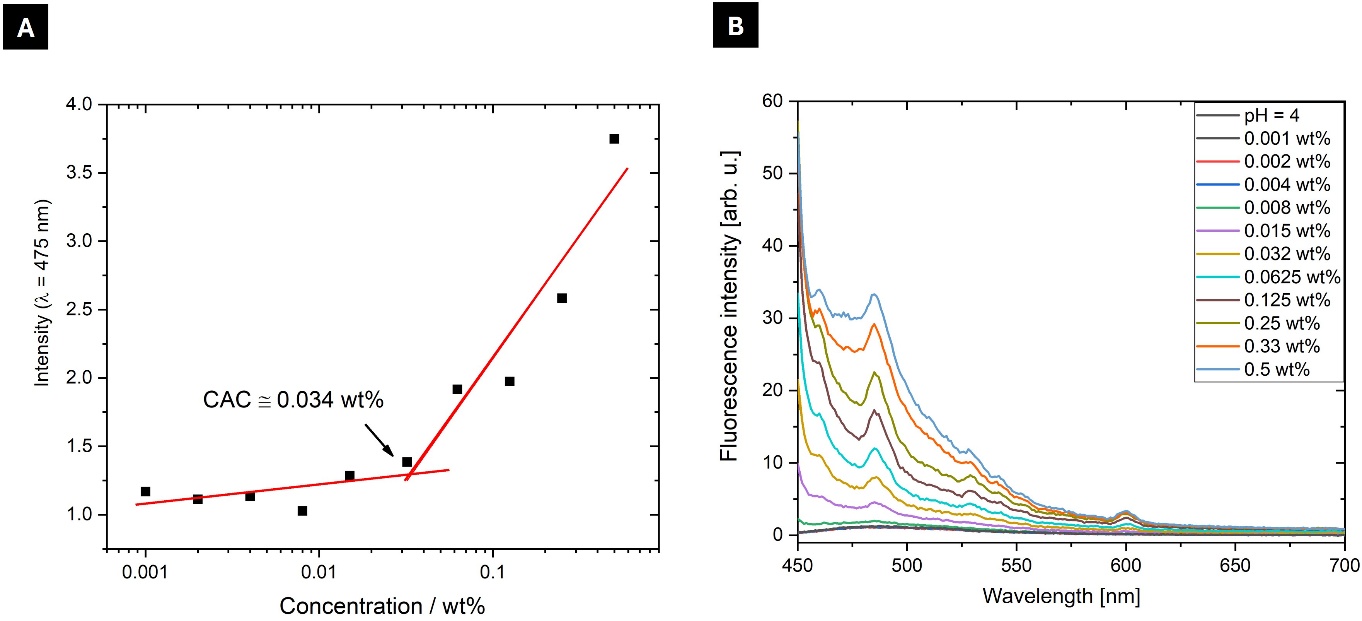


**SI Figure S5**. CAC from ThT fluorescence experiment for C_16_-RTTRS acetate salt, pH = 4. (A) CAC from fluorescence intensity at λ = 475 and (B) original spectra at lipopeptide concentrations indicated.


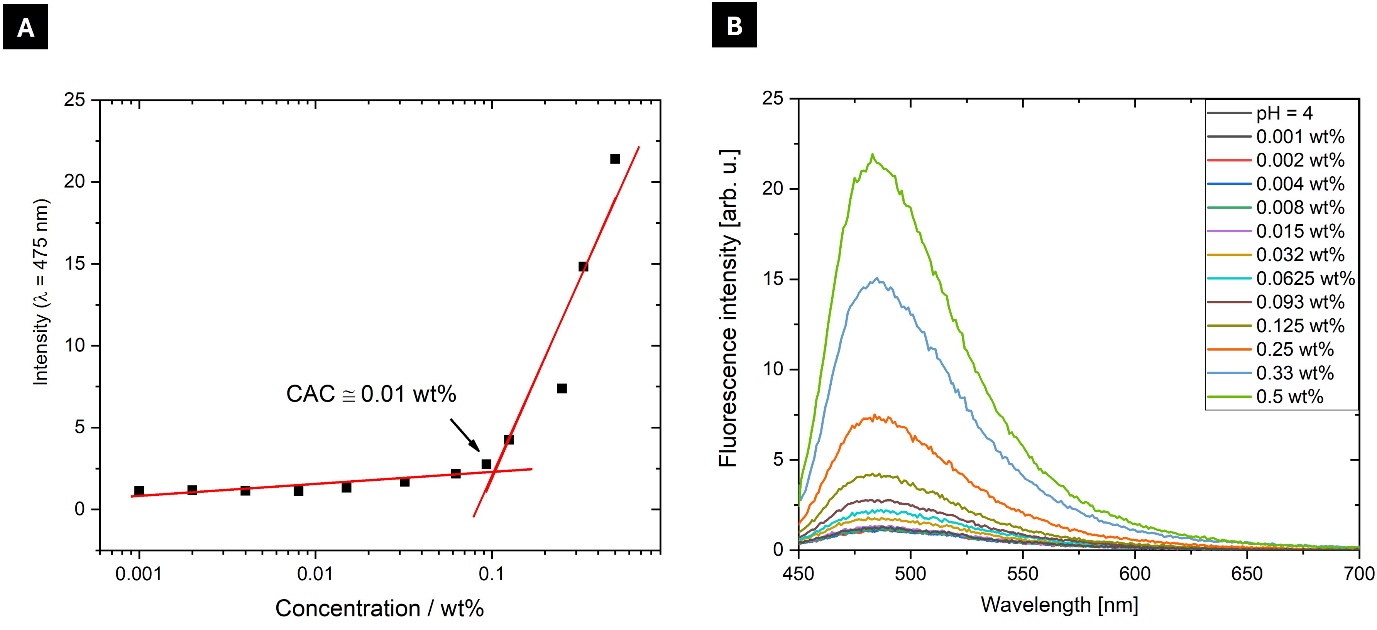


**SI Figure S6**. CAC from ThT fluorescence experiment for C_16_-RTTRS TFA salt, pH = 4. (A) CAC from fluorescence intensity at λ = 475 and (B) original spectra at lipopeptide concentrations indicated.


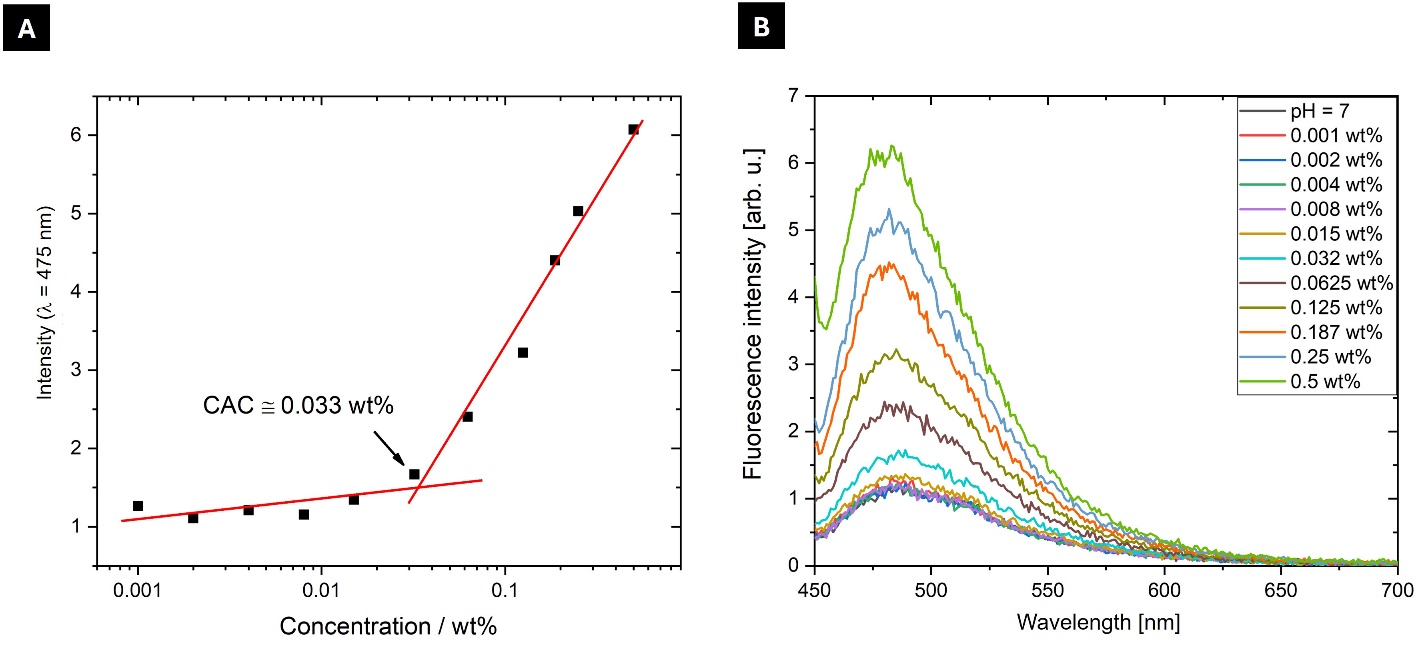


**SI Figure S7**. CAC from ThT fluorescence experiment for C_16_-RTTRS acetate salt, pH = 7. (A) CAC from fluorescence intensity at λ = 475 and (B) original spectra at lipopeptide concentrations indicated.


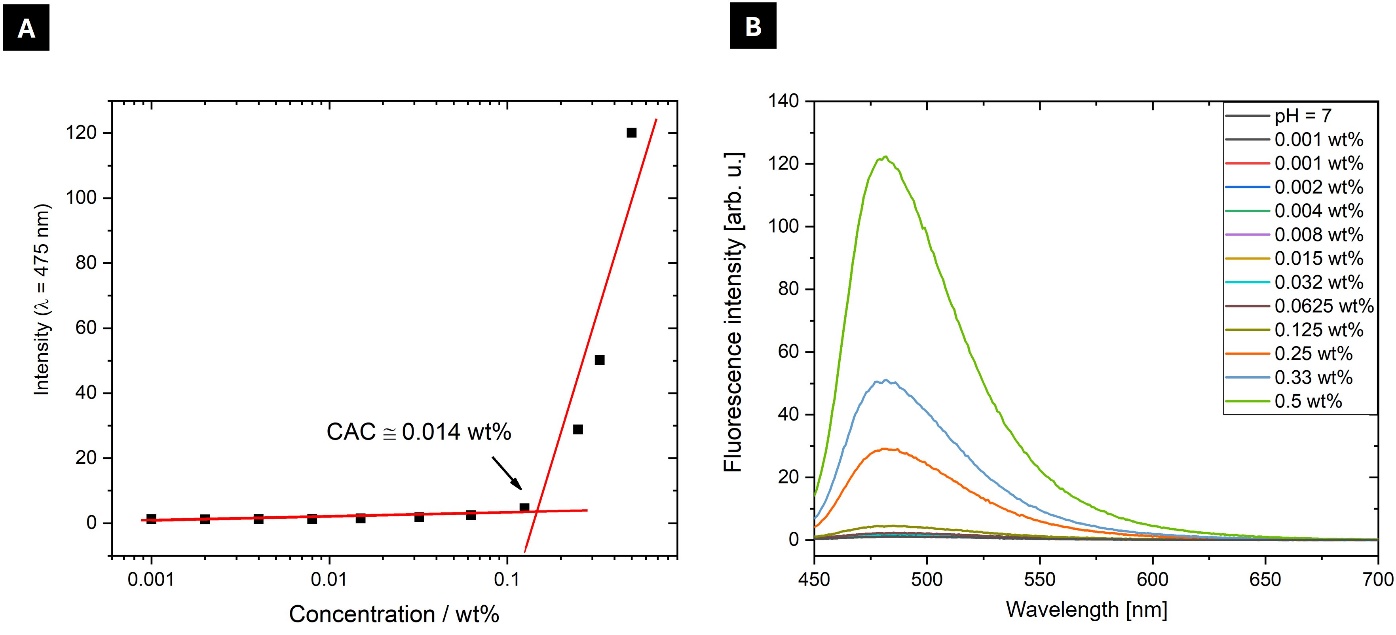
 **SI Figure S8**. CAC from ThT fluorescence experiment for C_16_-RTTRS TFA salt, pH = 7. (A) CAC from fluorescence intensity at λ = 475 and (B) original spectra at lipopeptide concentrations indicated.





**SI Fig. S9**. Additional cryo-TEM image for 1 wt% solution of C_16_-KTTKS (TFA) at pH 2





**SI Fig. S10**. Additional cryo-TEM image for 1 wt% solution of C_16_-KTTKS (acetate) at pH 2.





**SI Fig. S11**. Additional cryo-TEM image for 1 wt% solution of C_16_-KTTKS (TFA) at pH 4.





**SI Fig. S12**. Additional cryo-TEM image for 1 wt% solution of C_16_-KTTKS (acetate) at pH 4.





**SI Fig. S13**. Additional cryo-TEM image for 1 wt% solution of C_16_-KTTKS (TFA) at pH 7.





**SI Fig. S14**. Additional cryo-TEM image for 1 wt% solution of C_16_-KTTKS (acetate) at pH 7.

**

**

**SI Fig. S15**. Additional cryo-TEM image for 1 wt% solution of C_16_-RTTRS (TFA) at pH 2.





**SI Fig. S16**. Additional cryo-TEM image for 1 wt% solution of C_16_-RTTRS (acetate) at pH 2.

**

**

**SI Fig. S17**. Additional cryo-TEM image for 1 wt% solution of C_16_-RTTRS (TFA) at pH 5.





**SI Fig. S18**. Additional cryo-TEM image for 1 wt% solution of C_16_-RTTRS (acetate) at pH 5.





**SI Fig. S19**. Additional cryo-TEM image for 1 wt% solution of C_16_-RTTRS (TFA) at pH 7.





**SI Fig. S20**. Additional cryo-TEM image for 1 wt% solution of C_16_-RTTRS (acetate) at pH 7.

**SI Fig.S21**. SAXS data (open symbols) for 1 wt% solutions of C_16_-KTTKS at pH 2 or pH 7 with acetate or TFA along with model form factor fits (solid lines) described in the text. Fit parameters listed in SI Table S1.

**SI Fig.S22**. SAXS data (open symbols) for 1 wt% solutions of C_16_-RTTRS at pH 2 or pH 7 with acetate or TFA along with model form factor fits (solid lines) described in the text. Fit parameters listed in SI Table S2.

**Figure S23**. Dynamic shear moduli stress sweep curves for 0.5 (open dots and squares) or 2 wt% C_16_-KTTKS (squares and dots) hydrogels at a fixed frequency ω= 6.283 rad s^–1^.

**
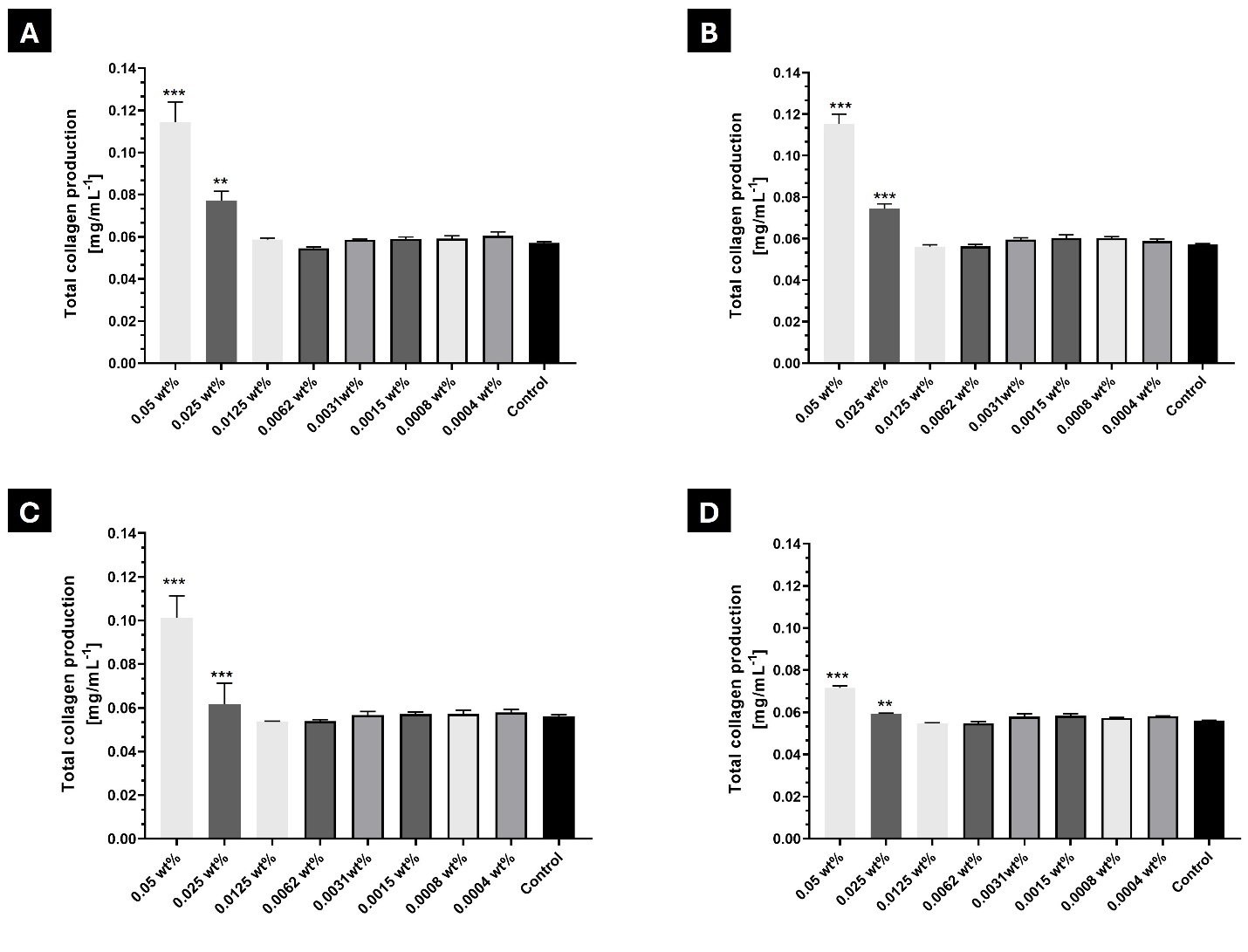
**

**Figure S24**. Total collagen production determined by the picrosirius red assay for (A) TFA salt of C_16-_KTTKS, (B) Acetate salt of C_16-_KTTKS, (C) TFA salt of C_16-_RTTRS and (D) Acetate salt of C_16-_RTTRS. ANOVA with Bonferroni correction for multiple comparisons, n = 3

**Table S1.** Parameters extracted from the fitting of the SAXS data for 1 wt% solutions of C_16_-KTTKS at pH values indicated.^a^

|  | **C_16_-KTTKS**  **acetate**  **pH 2**  *Core-shell sphere + Gaussian Bilayer* | **C_16_-KTTKS**  **TFA**  **pH 2**  *Core-shell sphere*^b^ | **C_16_-KTTKS**  **acetate**  **pH 7**  *Gaussian Bilayer + lamellar structure factor* | **C_16_-KTTKS**  **TFA**  **pH 7**  *Gaussian Bilayer + lamellar structure factor* |
| --- | --- | --- | --- | --- |
| *w*_1_ | 0.064 | N/A | N/A | N/A |
| *t* ± Δ*t* [Å] | 28.0 ± 2.0 | - | 35.9 ± 6.0 | 34.3 ± 4.2 |
| *ρ*_H_  [cm^-1^] | 1.74×10^-7^ | - | 3.23×10^-7^ | 3.31×10^-7^ |
| *σ*_H_ [Å] | 5.0 | - | 5.0 | 4.8 |
| *ρ*_C_ [cm^-1^] | 1.09 ×10^-7^ | - | 1.48×10^-8^ | 8.20×10^-8^ |
| *σ*_C_ [Å] | 5.0 | - | 6.9 | 29.7 |
| *D* [Å] | 770 | - | 496 | 433 |
| *N* | - | - | 8 | 8 |
| *d* | - | - | 52.0 | 52.0 |
| *η* | - | - | 0.01 | 0.01 |
| *ν* |  | - | 0.30 | 0.20 |
| *w*_2_ | 0.582 | N/A | N/A | N/A |
| *R*_out_ [Å] | 33.0 ± 0.01 | 32.0 ± 0.01 | - | - |
| *R*_in_ [Å] | 13.4 | 13.4 | - | - |
| *μ* | -2.64 | -2.29 | - | - |
| *ρ* [cm^-1^] | 3.21×10^-6^ | 3.08×10^-5^ | - | - |
| *C* | 1.20×10^-3^ | 1.24×10^-1^ | 2.00×10^-4^ | 4.41×10^-4^ |

^a^ Data fitted using the software SASfit.^1-2^

^b^ Only micelle form factor data included since data (previously reported^3^) covers high q only

**Key: Gaussian bilayer:** half layer thickness *t* (Gaussian polydispersity Δ*t*), scattering contrast of outer (headgroup) layers *ρ*_H_, and core (lipid chain) layer *ρ*_C_, Gaussian widths *σ*_C_ and *σ*_H_ of core and headgroup layers respectively, *D* diameter (width) of layer system (when *D* >> *t* as here, it acts as a scaling parameter for the form factor). **Lamellar structure factor (Caillé model**^4^**):** *N* number of layers, *d* layer spacing, *η* lamellar fluctuation parameter, *ν* diffuse scattering term. **Spherical Core-Shell:** *R*_out_ outer radius (Gaussian polydispersity), Rin inner (core) radius, *μ* ratio of scattering intensity of core, *ρ* scattering contrast of outer part with respect to solvent. **Background:** constant background, *C*. Weightings for two-component form factors, *w*_1_, *w*_2_.

**Table S2.** Parameters extracted from the fitting of the SAXS data for 1 wt% solutions of C_16_-RTTRS at pH values indicated.^a^

|  | **C_16_-RTTRS**  **acetate**  **pH 2**  *Core-shell sphere + Gaussian bilayer*^b^ | **C_16_-RTTRS**  **TFA**  **pH 2**  *Core-shell sphere + Gaussian bilayer* | **C_16_-RTTRS acetate**  **pH 7**  *Core-shell sphere + Gaussian bilayer*^b^ | **C_16_-RTTRS TFA**  **pH 7**  *Gaussian Bilayer + lamellar structure factor* |
| --- | --- | --- | --- | --- |
| *w*_1_ | 0.02 | 0.79 | 0.02 | N/A |
| *t* ± Δ*t* [Å] | 28.0 ± 2.0 | 30.0 ± 3.0 | 28.0 ± 2.0 | 29.6 ± 1.0 |
| *ρ*_H_  [cm^-1^] | 1.19×10^-7^ | 1.92×10^-7^ | 1.19×10^-7^ | 1.97×10^-7^ |
| *σ*_H_ [Å] | 5.0 | 5.0 | 5.0 | 7.5 |
| *ρ*_C_ [cm^-1^] | 1.00×10^-7^ | 1.00×10^-7^ | 1.00×10^-7^ | 2.38×10^-8^ |
| *σ*_C_ [Å] | 5.0 | 5.0 | 5.0 | 9.9 |
| *D* [Å] | 1000 | 471 | 1000 | 264 |
| *N* | - | - | - | 5 |
| *d* | - | - | - | 42.0 |
| *η* | - | - | - | 0.1 |
| *ν* |  | - | - | 24.0 |
| *w*_2_ | 0.85 | 0.91 | 0.90 | N/A |
| *R*_out_ [Å] | 34.0 ± 0.01 | 34.0 ± 0.01 | 34.0 ± 0.01 | - |
| *R*_in_ [Å] | 13.4 | 13.4 | 13.4 | - |
| *μ* | -2.64 | -2.40 | -2.64 | - |
| *ρ* [cm^-1^] | 3.4×10^-6^ | 3.1×10^-6^ | 3.4×10^-6^ | - |
| *C* | 1.30×10^-3^ | 1.00×10^-3^ | 1.50×10^-3^ | 1.00×10^-3^ |

^a^ Data fitted using the software SASfit.^1-2^

^b^ Most parameters fixed for the two fits

**Key: Gaussian bilayer:** half layer thickness *t* (Gaussian polydispersity Δ*t*), scattering contrast of outer (headgroup) layers *ρ*_H_, and core (lipid chain) layer *ρ*_C_, Gaussian widths *σ*_C_ and *σ*_H_ of core and headgroup layers respectively, *D* diameter (width) of layer system (when *D* >> *t* as here, it acts as a scaling parameter for the form factor). **Lamellar structure factor (Caillé model**^4^**):** *N* number of layers, *d* layer spacing, *η* lamellar fluctuation parameter, *ν* diffuse scattering term. **Spherical Core-Shell:** *R*_out_ outer radius (Gaussian polydispersity), Rin inner (core) radius, *μ* ratio of scattering intensity of core, *ρ* scattering contrast of outer part with respect to solvent. **Background:** constant background, *C*. Weightings for two-component form factors, *w*_1_, *w*_2_.

**References**

(1) Bressler, I.; Kohlbrecher, J.; Thünemann, A. F., SASfit: a tool for small-angle scattering data analysis using a library of analytical expressions. *J. Appl. Cryst.* **2015,** *48*, 1587–1598.

(2) Kohlbrecher, J.; Bressler, I., Updates in SASfit for fitting analytical expressions and numerical models to small-angle scattering patterns. *J. Appl. Cryst.* **2022,** *55*, 1677–1688.

(3) Dehsorkhi, A.; Castelletto, V.; Hamley , I. W.; Adamcik, J.; Mezzenga, R., The effect of pH on the self-assembly of a collagen derived peptide amphiphile. *Soft Matter* **2013,** *9*, 6033–6036.

(4) Caillé, A., X-Ray Scattering by Smectic-A Crystals. *C. R. Hebdo. Des Seances Acad. Sci. B* **1972,** *274* (14), 891–893.
